# Afatinib or Bevacizumab in combination with Osimertinib efficiently control tumor development in orthotopic murine models of non-small lung cancer

**DOI:** 10.1371/journal.pone.0304914

**Published:** 2024-06-27

**Authors:** Ulrich Jarry, Megane Bostoen, Jérome Archambeau, Raphaël Pineau, Laura Chaillot, Florence Jouan, Hélène Solhi, Hugo Ferrari, Rémy Le Guevel, Valentine Mennessier, Hervé Lena, Romain Corre, Charles Ricordel, Thierry Guillaudeux, Rémy Pedeux

**Affiliations:** 1 Univ Rennes, CNRS, INSERM, BIOSIT UAR 3480, US_S 018, Oncotrial, Rennes, France; 2 Biotrial Pharmacology, Unité de Pharmacologie Préclinique, Rennes, France; 3 Univ Rennes, INSERM, OSS (Oncogenesis Stress Signaling), UMR_S 1242, CLCC Eugene Marquis, Rennes, France; 4 Centre Hospitalier Universitaire de Rennes, Univ Rennes, Rennes, France; Osmania University, Hyderabad, India, INDIA

## Abstract

Lung cancer is one of the most common and deadliest cancers. Preclinical models are essential to study new therapies and combinations taking tumor genetics into account. We have established cell lines expressing the luciferase gene from lines with varied genetic backgrounds, commonly encountered in patients with pulmonary adenocarcinoma. We have characterized these lines by testing their response to multiple drugs. Thus, we have developed orthotopic preclinical mouse models of NSCLC with very high engraftment efficiency. These models allow the easy monitoring of tumor growth, particularly in response to treatment, and of tumor cells dissemination in the body. We show that concomitant treatment with osimertinib (3rd generation tyrosine kinase inhibitor targeting mutated EGFR) and bevacizumab (anti-angiogenic targeting VEGF) can have a beneficial therapeutic effect on EGFR-mutated tumors. We also show that the addition of afatinib to osimertinib-treated tumors in escape leads to tumor growth inhibition. No such effect is observed with selumetinib or simvastatin. These preclinical mouse models therefore make it possible to test innovative therapeutic combinations and are also a tool of choice for studying resistance mechanisms.

## Introduction

Lung cancer is one of the most common and deadly cancers in Europe and worldwide [1]. Of these, nearly 85% are classified as non-small cell lung cancer (NSCLC) with more than 50% of the patients diagnosed at an advanced stage [2]. Current treatments, based mainly on platinum-based chemotherapy, radiotherapy, and surgery, are poorly effective, with a median survival time of no more than 10 months [3].

In the past two decades, several oncogenic factors have been identified in NSCLC patients, such as EGFR mutations and ALK rearrangements, leading to personalized therapies. Regarding EGFR mutations, mainly identified as exon 19 deletion [del 19] and exon 21 point mutation L858R, they are present in about 15% of patients of European or Caucasian origin and 35% of Asian patients [4, 5]. These mutations were associated with 70% - 80% of responses to epidermal growth factor receptor (EGFR) tyrosine kinase inhibitors (TKI) in patients treated with gefitinib, the first FDA-approved oral EFGR TKI [6]. Subsequently, the first and second generation EFGR TKI (e.g., gefitinib, erlotinib, afatinib) have been successfully used and recommended as first-line therapies for NSCLC patients with activating EGFR mutations [7]. However, the use of these EGFR TKI has been associated with the emergence of resistance mechanisms after 9 to 13 months of treatment [8, 9]. Among them, the T790M mutation is the most common mechanism observed in response to erlotinib, gefitinib and afatinib [9]. More recently, the 3rd generation of EFGR TKI (e.g. osimertinib), effective for NSCLC patients with the EGFR T790M mutation has achieved strong and durable responses, as demonstrated in the phase 2 AURA2 trial [10]. Osimertinib is now recommended as first-line therapy for patients with EGFR-mutated NSCLC [11]. However, other resistance mechanisms also occur in response to osimertinib, such as EGFR mutations G796/C797, L792, and L718/G719, downstream activated oncogenes such as MET, KRAS, and PIK3CA, or high MET amplification [12].

Since 2006, the US FDA has approved bevacizumab in combination with carboplatin and paclitaxel as a first-line treatment for advanced NSCLCs [4]. Bevacizumab is a monoclonal antibody that selectively targets vascular endothelial growth factor VEFG and subsequently angiogenesis mechanisms that play a crucial role in lung cancer incidence, progression, and metastasis [13]. Bevacizumab (Avastin) is also used in other tumor indications such as colon and rectal metastatic carcinoma, breast cancer, and glioblastoma [6, 7]. In NSCLC, although the addition of bevacizumab to standard chemotherapies has improved median overall and progression-free survival [3], significant progress remains to be made.

In this study, we examined the antitumor efficacy of osimertinib combination with afatinib or bevacizumab in orthotopic mouse models of human NSCLC tumors. We used four NSCLC cell lines (A549, PC9, H1975 and HCC827 cell lines), associated with different EGFR mutations. These cell lines were first engineered to express luciferase (Luc^+^) and then their response profiles to several chemotherapeutic agents were determined by EC50 and CFU assays. Luc^+^ cells were used to establish orthotopic models, as previously demonstrated [14] and finally, combinations of osimertinib plus afatinib or osimertinib plus bevacizumab were evaluated. The results showed control of tumor progression and/or regression in response to these treatments, underscoring the value of these combinations as promising and effective approaches for the treatment of NSCLC.

## Materials and methods

### Human NSCLC tumor cells and luciferase expression

The A549 cell line (CCL-185^TM^, ATCC, Manassas, VA) was cultured in low-glucose DMEM (Dutscher, Brumath, France) supplemented with 10% heat-inactivated FBS (Dutscher) and 2 mM L-glutamine (PanBiotech, Aidenbach, Bavaria, Germany). PC-9 (formely known as PC-14; ACACC90071810; Sigma-Aldrich, St. Louis, MO) was cultured in RPMI (Dutscher) supplemented with 10% heat-inactivated FBS and 2 mM L-glutamine. H1975 (CRL-5908^TM^, ATCC) and HCC827 (CRL-2868^TM^, ATCC) cell lines were cultured in RPMI supplemented with 10% heat-inactivated FBS, 2 mM L-glutamine, 10 mM HEPES (Gibco, Carlbad, CA), 1 mM sodium pyruvate (Gibco) and 0.15% sodium bicarbonate (Gibco). For luciferase expression, A549 and H1975 cells were transduced with RediFect Red-Fluc-Puromycin lentiviral particles (PerkinElmer, Waltham, MA) whereas PC9 and HCC827 cells were transfected with pGL4.51 vector [luc2/CMV/NEO] (Promega, Madison, WI), according to the manufacturer’s instructions.

### Drugs

The EGFR TKI Osimertinib, Afatinib and Erlotinib, the topoisomerase II inhibitors Etoposide and Doxorubicin, the microtubule polymer stabilizer Paclitaxel, the platinum salts Cisplatin and Carboplatin and the DNA/RNA synthesis inhibitor Fluorouracil (5-FU) were obtained from Interchim (Montluçon, France). HDAC inhibitors Panobinostat, Vorinostat, Belinostat, Droxinostat and RGFP966, the C-Met and ALK inhibitor Crizotinib, the ATM inhibitors AZD0156 and KU55933, the ATR inhibitor AZD6738, the IRE1α endoribonuclease domain inhibitor MKC-3946, the PARP1/2 inhibitor Olaparib, and the Wee1 inhibitor MK1775 were obtained from Selleckchem (Houston, TX). Bevacizumab was kindly provided by Genentech (South San Francisco, CA).

### EC50 determination tests

Chemicals are solubilized in DMSO at a concentration of 10 mM (stock solution) and diluted in culture medium to the desired final concentrations. The dose effect cytotoxic assays (EC50 determination) is performed by increasing concentrations of each chemical (final well concentrations: 0.1 μM– 0.3 μM– 0.9 μM– 3 μM– 9 μM—25μM). Cells are plated in 96 wells plates (4000 cells/well). Twenty-four hours after seeding, cells are exposed to chemicals. After 48h of treatment, cells are washed in PBS and fixed in cooled 90% ethanol/5% acetic acid for 20 minutes and the nuclei are stained with Hoechst 33342 (B2261 Sigma). Image acquisition and analysis are performed using a Cellomics ArrayScan VTI/HCS Reader (ThermoScientific). The survival percentages are calculated as the percentage of cell number after compound treatment over cell number after DMSO treatment. The relative EC50 are calculated using the curve fitting XLfit 5.5.0.5 (idbs) integrated in Microsoft Excel as an add on. The 4 Parameter Logistic Model or Sigmoidal Dose-Response Model is used (fit = (A+((B-A)/(1+((C/x)^D))))).

### Colony formation assay

The cells were treated or not, with Etoposide, Erlotinib, Afatinib or Osimertinib for 24 h either at the doses of EC50, EC50/10 or EC50x10 as determined previously. Cells were then collected, plated into twelve-well plates and incubated in a 5% CO2 incubator at 37°C for 7–11 days (corresponding to 5 doubling times). Cells were then fixed with 4% paraformaldehyde-PBS, stained with Eosin (Sigma-Aldrich) and washed with distillated water before colony counting.

### Immunodeficient mice, implantation and treatment of tumor cells

Female and male NSG (*NOD*.*Cg-Prkdcscid Il2rgtm1Wjl/SzJ)* mice were obtained from Charles River Laboratories (Wilmington, MA). Mice were bred in the Rennes University animal facility (Arche / UAR BIOSIT / Rennes 1 University) under SPF status and used at 6 to 12 weeks of age, in accordance with institutional guidelines. Animal experiments have been approved in advance by the ethics committee (CEEA– 007 Comité rennais d’éthique en matière d’expérimentation animale, Approval # 8887). Health reports indicated that the mice were free of known viral, bacterial and parasitic pathogens. Mice were acclimated at least 7 days before use. Animals were housed with a 12 hours day-night cycle, with lights on at 8:00 am, at temperature (22±1°C), with free access to food and water in filter cages (Tecniplast, Bugiggiate, Italy) enriched with a mouse house (3–5 mice per cage). The animals’ health status was monitored throughout the experiments by a health monitoring program according to Federation of European Laboratory Animal Science Associations (FELASA) guidelines. Throughout the experiments, a weight loss greater than 10% was the criterion for euthanizing the animals. According to the critical phase of the experiments, following the instructions of the animal welfare structure, animals’ weight was measured once, twice or three times a week and the occurrence of severe clinical signs was monitored at the same time. Monitoring of animal welfare (weight curves) was checked independently once a week by the members of the animal welfare structure. At the end of the experiment, mice were euthanized by cervical dislocation. Mice were free of all viral, bacterial, and parasitic pathogens listed in the FELASA recommendations. Each mouse was identified by a number, which avoided confusion during subsequent treatments. For orthotopic implantation, mice were injected intravenously with 1x10^6^ tumor cells in 100 μL sterile PBS. Tumor nests were assessed by bioluminescence, allowing the inclusion and the randomization of mice in the study. Randomization was performed by the study leaders (UJ & RP). It should be noted that no mouse was excluded from the study. Throughout the experiments, animals didn’t show any severe clinical signs. Tumor growth was assessed by bioluminescence.

For this study 217 mice were used, each defined as an experimental unit: A) 20 NSG for the orthotopic model set up, 5 for each tumor cell line, B) 86 NSG for the treatment assay regarding the effect Osimertinib and Erlotinib (5 untreated, 6 Osimertinib 5 mg/kg and 6 Erlotinib 50 mg/kg for model A549, 7 untreated, 7 Osimertinib 1 mg/kg, 7 Osimertinib 5 mg/kg, 7 Erlotinib 12.5 mg/kg, 7 Erlotinib 25 mg/kg and 7 Erlotinib 50 mg/kg for model PC9, 6 untreated, 7 Osimertinib 1 mg/kg, 7 Osimertinib 5 mg/kg, and 7 Erlotinib 50 mg/kg for model H1975, C) 48 NSG for the treatment assay regarding the effect of combining Osimertinib with Bevacizumab (3 untreated, 3 Osimertinib 1 mg/kg, 5 Bevacizumab 5mg/kg and 5 Osimertinib *plus* Bevacizumab combination for each of the A549, PC9 and H1975 models), and D) 21 NSG for the treatment assay for the combination of Osimertinib with Afatinib (10mg/kg), Selutinib (50mg/kg) and Simvastatin (20mg/kg) in PC9 model (7 mice per groups). Osimertinib (5 days a week) and bevacizumab (2 days a week) were administered intraperitoneally (ip) in sterile PBS solution, whereas erlotinib (5 days a week), afatinib (2 days a week), selumetinib (2 days a week) and simvastatin (2 days a week) were administrated *per os* in 2% carboxymethylcellulose solution. Sample sizes were defined as sufficient for statistical analysis.

### Bioluminescence assays

For *in vitro* Bioluminescence assays, cells were plated at different numbers in a 96 wells plate for 4 h. Then, 20 μL of D-Luciferin K^+^ solution (Interchim) at 15 μg/μL was distributed in each well and Bioluminescence signals were monitored using the Photon imager (Biospace Lab, Nesles la Vallée, France) equipped with a highly sensitive cooled CCD camera. For *in vivo* Bioluminescence assays, mice were injected with 150 μL of 15 μg/μL D-Luciferin K^+^ solution, anesthetized via inhaled isoflurane and monitored using the Photon imager. Data were analyzed using cpm (counts per minute) per cm^2^ for *in vitro* assays and, for *in vivo* assays, either on the chest (to analyze the lungs) or on the whole body. The bioluminescence images showed in the figures are not indicative of the volume occupied by tumor cells in the body by comparison to the body of mice. When the mice are euthanized, the autopsy does not locate the tumors because they are microscopic. It takes a targeted method like IHC to see the tumor cells. All the images shown in the manuscript were taken in saturated conditions so as to be able to easily identify the localization and dissemination of tumor cells.

### Histochemistry analysis

Lungs were fixed with 4% paraformaldehyde-PBS, embedded in paraffin wax and serially sectioned (4 μm). Sections were then stained with Hematoxylin and eosin (H&E) or with anti-B220 (BD Biosicience, Franklin Lakes, NJ), anti-CD31 (Dianova, Biozol Diagnostica Vertrieb GmbH, Eching, Germany) or anti-Phospho-ERK (Cell Signaling Technology, Danvers, MA) mAb associated to a revelation based on diaminobenzidine (DAB). Finally, slides were scanned using the NanoZoomer 2.0 HT (Hamamatsu Photonics K.K., Hamamatsu, Japan).

### Statistical analysis

Data are expressed as mean ± SEM and were analyzed using GraphPad Prism 7.0 software (GraphPad Software, Inc., San Diego, CA). *In vitro* assays were analyzed using Student’s *t* (*p<0.05; **p<0.005; ***p< 0.0005) and *in vivo* Bioluminescence assays were analyzed using F tests of equal variances to reveal significant differences.

## Results

### Generation of Luc^+^ cell lines of NSCLC

**Fig 1:**
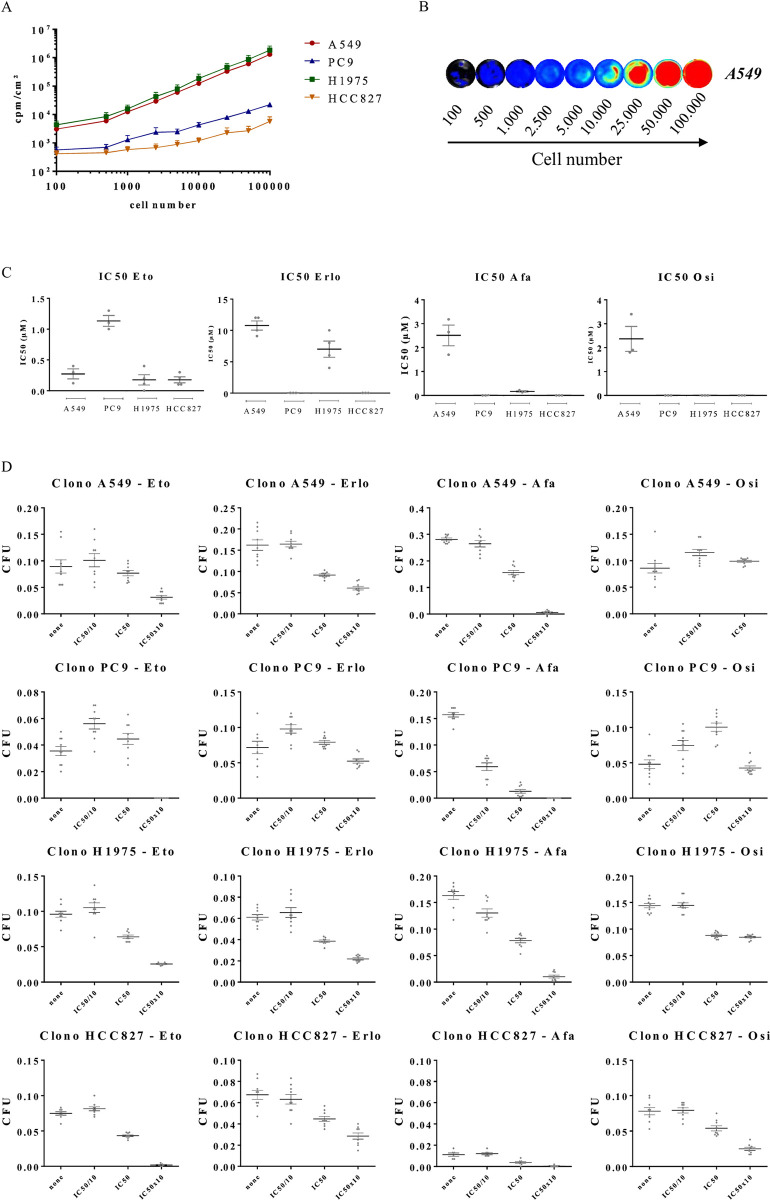
Generation and characterization of Luc^+^ NSCLC cell lines. The human NSCLC A549, H1975, PC9 and HCC827 were modified for the expression of Luciferase. Cells were plated at different quantities and bioluminescence was assessed. **(A)** The results are expressed as cpm/cm^2^ (mean ± SEM, n = 3). **(B)** Picture shows **a** representative result of bioluminescence intensity. **(C).** Luc^+^ NSCLC cells were treated with different concentrations of etoposide (Eto), erlotinib (Erlo), afatinib (Afa) and osimertinib (Osi), and then used for EC50 assays. Results are expressed as the EC50 (n = 6). **(D)** Luc^+^ NSCLC cells were treated with etoposide, erlotinib, afatinib and osimertinib at the EC50 concentration (EC50), or 10x less (EC50/10) or 10x higher (EC50x10), and then used for CFU assays (n = 6 or more).

To consider the different human EGFR mutation profiles observed in NSCLC patients, four tumor cell lines with different EGFR mutations were used: (i) A549 cells that have KRAS G12S mutation and no EGFR mutation, (ii) PC9 cells that have exon 19 deletion, (iii) H1975 cells that have T790M and L858R mutations, and (iv) HCC827 cells that have exon 19 deletion. Thus, A549 cells would be resistant while PC9 and HCC827 cells would be sensitive to EGFR TKI, and H1975 cells would be resistant to both 1st and 2nd generation EGFR TKI but not to 3rd generation Osimertinib. Stable luciferase-expressing cell lines were established using either the RediFect-Fluc-Puromicin lentivector (A549 and H1975 cells) or the pGL4.51 [Luc2/CMV/Neo] plasmid (PC9 and HCC827 cells) in order to monitor tumor progression in vivo. The bioluminescence intensities of each Luc^+^ cell line were evaluated *in vitro* (**Fig 1A and 1B**). The results show a linear proportionality between cell number and luminescence intensity between 500 and 100 000 cells. Interestingly, the bioluminescence signals were more intense when luciferase was induced using the RediFect-Fluc-Puromicin lentivector than the pGL4.51 [Luc2/CMV/Neo] plasmid. Genome stability between the parental and Luc^+^ lines was verified by STR characterization of the established Luc^+^ cell line (**S1A Fig**). While Luc^+^ H1975 cells showed little difference from wt H1975 cells (93% match with ATCC database), no difference was observed between wt and Luc^+^ A549, PC9 and HCC827 cell lines.

The cell lines were then characterized by analyzing the dose-response profiles of NSCLC cell lines (wt and Luc^+^) against a large number of drugs (**Fig 1C and 1D, S1B–S1D, S2 Figs**). Wt and Luc^+^ cells were treated with increasing concentrations of chemotherapeutic molecules for 48 h and the number of live cells was determined. The results were analyzed and used to determine both EC50s (**Fig 1C, S1C and S1D, S2A Figs, and S1 Table**) and the percentage of the minimum number of live cells with higher doses (Min) (**S1D, S2B Figs and S2 Table**). EC50 assays showed no significant difference between wt and Luc^+^ cells with either the EGFR TKI erlotinib, afatinib, and osimertinib or the topoisomerase II inhibitor etoposide. As expected, A549 cells are not sensitive to erlotinib, afatinib, or osimertinib; whereas, PC9 and HCC827 cells are sensitive to all three inhibitors. H1975 cells are resistant to erlotinib, mildly sensitive to afatinib and highly sensitive to osimertinib. Of note, as demonstrated by the minimum number of live cells with higher doses (Min), a large proportion of wt and Luc^+^ PC9 and H1975 cells were unaffected by osimertinib, afatinib, or erlotinib treatments (**S1D Fig and S2 Table**), suggesting that several subpopulations with different resistance properties may exist.

No significant differences were observed between wt and Luc^+^ cells, either in their EC50 or in the percentage of live cells at high doses, in response to the topoisomerase II inhibitor doxorubicin, the platinum salts cisplatin and carboplatin, or the DNA/RNA synthesis inhibitor fluorouracil (5-FU), the HDAC inhibitors panobinostat, vorinostat, belinostat, droxinostat and RGFP966, the C-Met or ALK inhibitor crizotinib, the ATM inhibitors AZD0156 and KU55933 the ATR inhibitor AZD6738, the endoribonuclease domain inhibitor IRE1α MKC-3946, the PARP inhibitor olaparib, and the Wee1 inhibitor MK1775 (**S1D, S3 Figs and S1, S2 Tables**).

To further characterize cells in response to treatments, colony formation assays were also performed in response to etoposide or the EGFR TKI erlotinib, afatinib, and osimertinib (**Fig 1D, S1B and S2B Figs**). The clonogenic test is an *in vitro* cell survival test based on the ability of a single cell to grow into a colony. It tests the ability of each cell in the population to undergo "unlimited" division, and thus assesses the remaining number of persistent cells that may be responsible for relapse/resistance. Wt and Luc^+^ cells were treated with either the doses corresponding to the previously determined EC50 or 10-fold less (EC50/10) or 10-fold more (EC50x10). Interestingly, no major differences were observed between wt and Luc^+^ NSCLC cell lines in drug response profiles.

Collectively, these data showed that the generated Luc^+^ cells were highly similar to the parental cells in terms of STR profiles and response to therapeutic molecules, demonstrating that they are robust NSCLC cell models for *in vivo* model development.

### Development of orthotopic *in vivo* models of NSCLC

**Fig 2:**
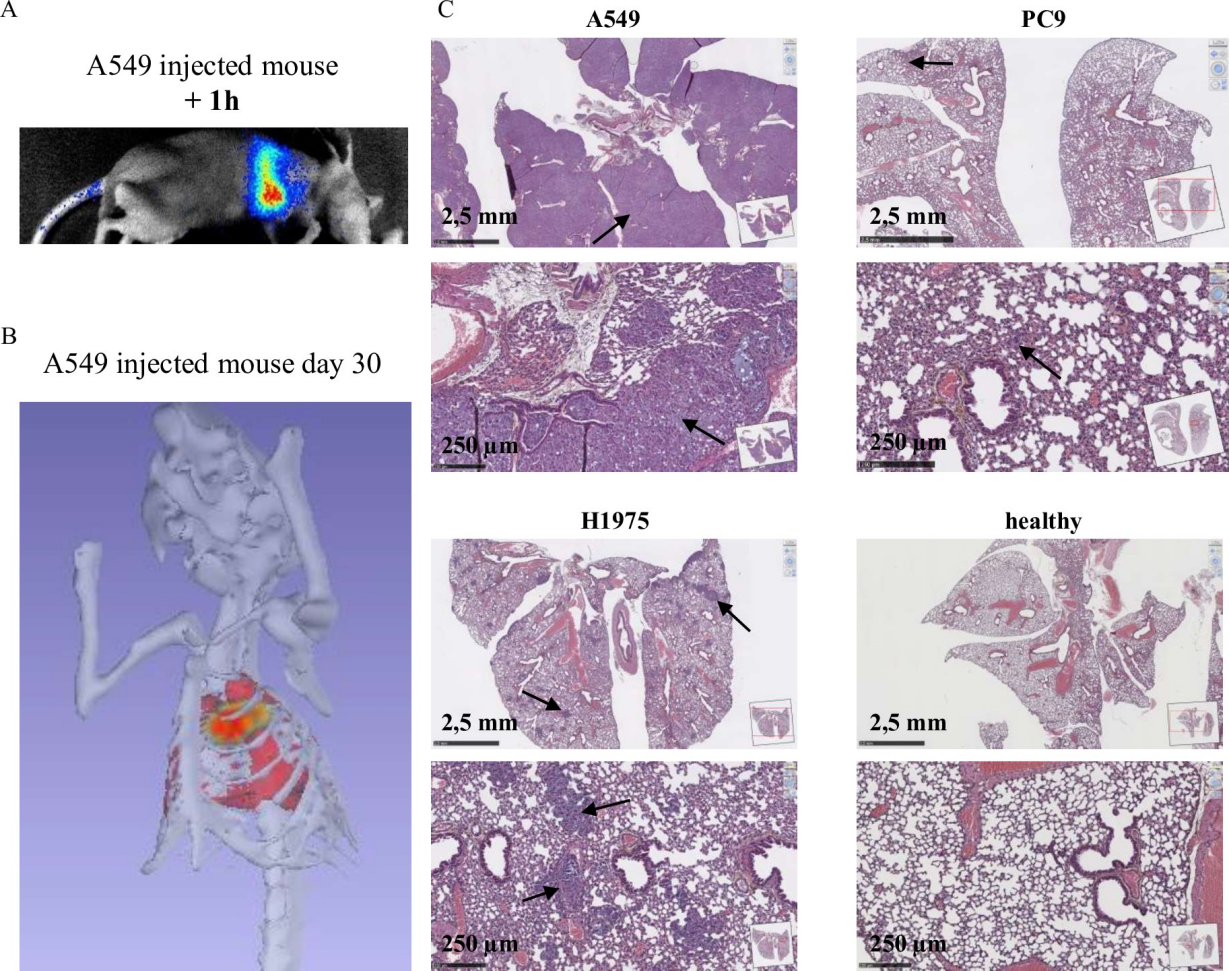
Set up of orthotopic models of NSCLC tumors in mice. Luc^+^ A549, PC9 and H1975 were injected in the tail vein of NSG mice. **(A)** Representative result of bioluminescence of tumor cells localization assessed 1 h after injection. **(B)** Representative result of tumor localization assessed using 4D module of BioImager and 3D Atlas reconstitution. **(C)** H&S coloration of lung sections of NSCLC tumor bearing mice. Healthy mice were used as control.

Luc^+^ lines were injected intravenously (iv) into the tail vein as previously described [14] to generate orthotopic tumors. Initial testing was performed using Balb/c nude mice and then Nod/Scid mice. However, tumor growth were not observed. Consequently, injections were performed in NSG (NOD.Cg-Prkdcscid Il2rgtm1Wjl/SzJ) mice, one of the most immunodeficient mouse strains. Interestingly, tumor growth was observed for all mice injected with Luc^+^ A549, Luc^+^ PC9 and Luc^+^ H1975 (**Fig 2A–2C**). Tumor cells’ localization was assessed by bioluminescence 1 h after injection. As shown in the representative image obtained using A549 cells (**Fig 2A**), they were mainly localized in the lungs. Tumor localization was also assessed during tumor development by bioluminescence and in particular with the 4 Views module of the Photon imager associated with a 3D reconstruction and an anatomical atlas. As shown in a representative image (**Fig 2B**) and video (**S4 Fig**) obtained by imaging an A549 tumor-bearing mouse 30 days after tumor implantation. Tumors were primarily observed in the lungs. Additional analysis, performed on lung sections from tumor-bearing mice by H&E staining, confirms tumor localization of Luc^+^ A549, Luc^+^ PC9, and Luc^+^ H1975 cells in the lung (**Fig 2C**). Of note, while Luc^+^ A549, Luc^+^ PC9, and Luc^+^ H1975 cells are suitable for iv-induced lung tumors in NSG no tumor was detected with Luc^+^ HCC827 cells.

### Evaluation of the therapeutic effects of Osimertinib in orthotopic models of NSCLC

**Fig 3:**
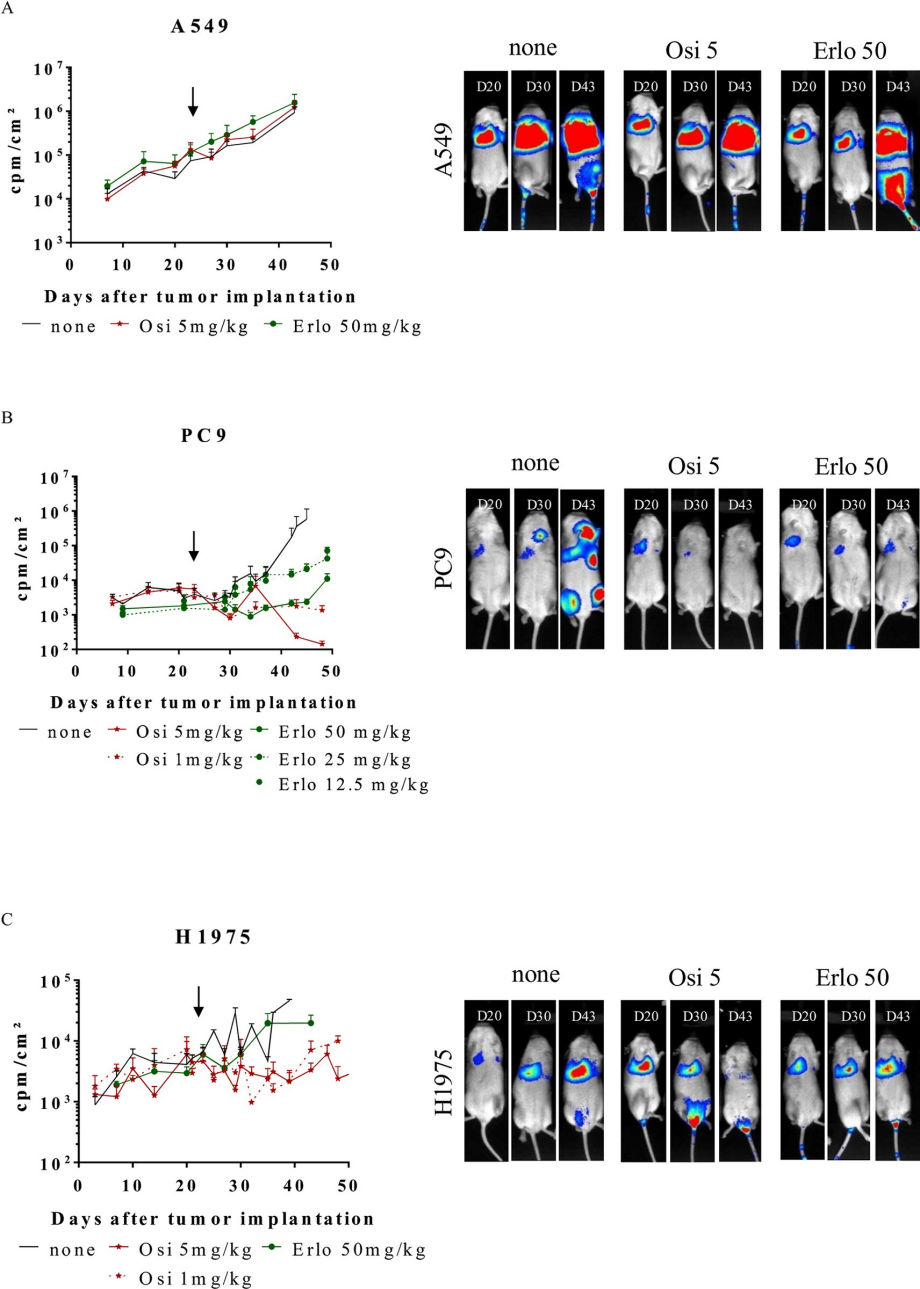
Osimertinib and Erlotinib therapeutic effects on tumor growth in orthotopic Luc^+^ NSCLC models in NSG mice. Tumor bearing mice (A549, PC9 and H1975) were treated, or not (▬), 5 days a week with osimertinib at 1 (---★---) or 5 (▬★▬) mg/kg or erlotinib at 12.5 (---▯---), 25 (---▯---) or 50 (▬▯▬) mg/kg. Tumor growth was assessed by bioluminescence. **(A)** The results are expressed as cpm/cm^2^ for the thorax area (mean ± SEM, n = 9)**. (B)** Pictures show representative results for bioluminescence of Luc^+^ A549, PC9 and H1975 tumor bearing mice untreated or treated with osimertinib 5 (Osi 5) mg/kg or erlotinib 50 (Erlo 50) mg/kg. See S7 Fig for statistical analysis.

The next step of this study was to evaluate the therapeutic effects of the EGFR TKI, osimertinib, in the three NSCLC tumor models Luc^+^ A549, Luc^+^ PC9 and Luc^+^ H1975. We first compared the effect of osimertinib and erlotinib on Luc^+^ A549, Luc^+^ PC9 and Luc^+^ H1975 tumor-bearing mice treated (**Fig 3 and S5–S8 Figs**). The treatment began when the signal emitted by the tumor at the thoracic level reached 10^3^ cpm/cm^2^ (PC9, H1975) or 10^4^ cpm/cm^2^ (A549) on average for the cohort of mice. Several doses were tested in these models, including for osimertinib the dose of 1 mg/kg. Tumor evolution was evaluated longitudinally by bioluminescence after injection. The results are expressed for the thorax area only (**Fig 3 and S5 Fig)**, or for the whole body (**S6 and S7 Figs**). The **Fig 3A** and **S6A Fig** show the mean ± SEM of bioluminescence intensities (express as cpm/cm^2^) while the **S4** and **S6 Figs** show the evolution of bioluminescence intensities for each mouse. The **Fig 3B** and **S5B Fig** show representative pictures of bioluminescence acquisitions. Neither high dose of erlotinib (50 mg/kg) nor osimertinib (5 mg/kg) affect tumor growth for Luc^+^ A549 tumor-bearing mice (**Fig 3A and 3B upper panels, S5 Fig upper panels, S6A Fig left panel and S7 Fig upper panels).** On the contrary mice carrying Luc^+^ PC9 tumors, erlotinib and osimertinib lead to a control of tumor growth (**Fig 3A and 3B middle panels, S5 Fig middle panel, S6A Fig middle panel, S6B and S7 Figs middle panel**). Tumor growth inhibition (TGI) is more pronounced with 50 mg/kg erlotinib compared to 12.5 and 25 mg/kg erlotinib (no difference was shown between these two conditions). Similarly, the effect of osimertinib at 5 mg/kg is more effective than at 1 mg/kg leading to a reduction in tumor bioluminescence intensity. Finally, regarding Luc^+^ H1975 tumor-bearing mice, high-dose erlotinib slows but does not inhibit tumor growth, while osimertinib allows TGI (**Fig 3A and 3B lower panels, S5 Fig lower panel, S6A Fig right panel, S6C and S7 Figs lower panel**) and long term TGI was more effective using osimertinib at 5 mg/kg compared to 1 mg/kg. Collectively, these data demonstrate that A549 cells are resistant to erlotinib and osimertinib, while PC9 cells are sensitive to both inhibitors and H1975 cells are resistant to erlotinib but sensitive to osimertinib.

### Therapeutic effects of osimertinib plus bevacizumab in orthotopic models of NSCLC

**Fig 4:**
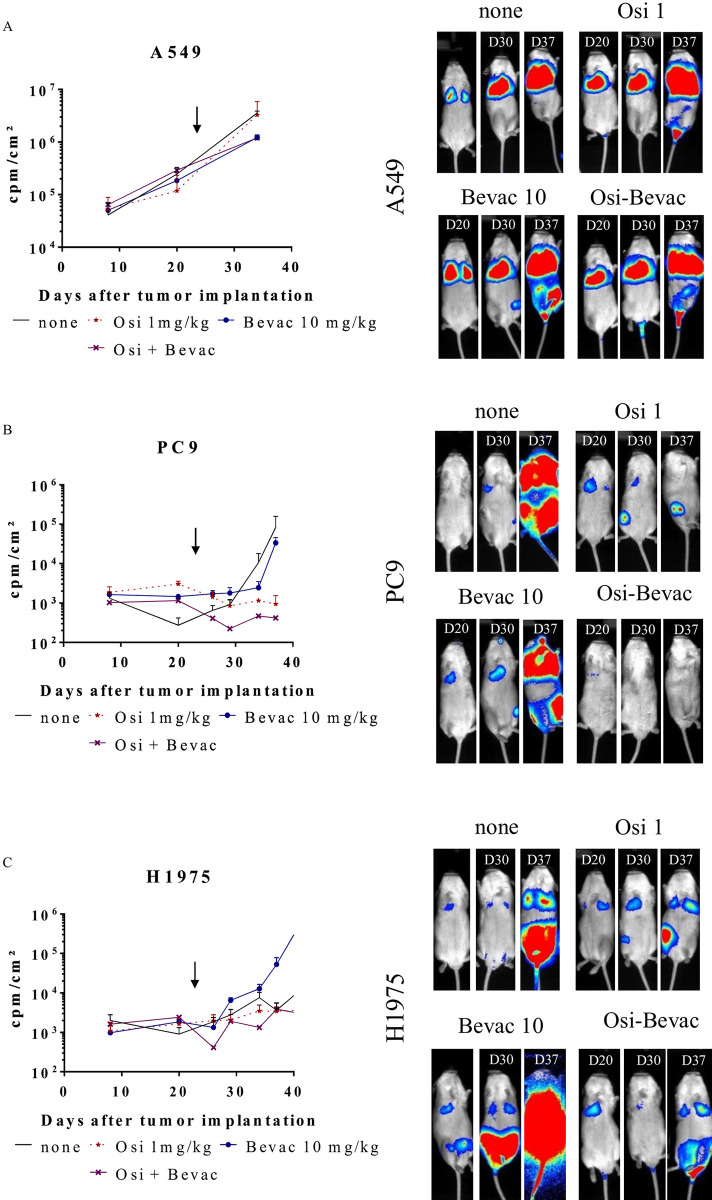
Osimertinib *plus* Bevacizumab therapeutic effects on tumor growth in orthotopic Luc^+^ NSCLC models in NSG mice. Tumor bearing mice (A549, PC9 and H1975) were treated, or not (▬), 5 days a week with osimertinib at 1 mg/kg (---★---), or 2 days a week with bevacizumab (▬▯▬), or both (▬X▬). Tumor growth was assessed by bioluminescence. **(A)** The results are expressed as cpm/cm^2^ (mean ± SEM, n = 5)**. (B)** Pictures show representative results for bioluminescence of Luc^+^ A549, PC9 and H1975 tumor bearing mice untreated or treated with osimertinib (Osi 1mg), bevacizumab (Bevac 5mg) or both (Osi-Bevac). See S7 Fig for statistical analysis.

Because combination of Bevacizumab with EGFR TK inhibitors showed improved tumor response, we assessed the combined effect of osimertinib *plus* bevacizumab in these three tumor models of NSCLC (**Fig 4 and S8–S10 Figs**). In this context, the osimertinib dose was 1 mg/kg and the bevacizumab dose was 10 mg/kg. As described previously, tumor growth was evaluated by bioluminescence over time after injection of tumor cells. The results are expressed for the thoracic zone (**Fig 4 and S9 Fig)**, or for the whole body (**S10 Fig**). **Fig 4A** shows the mean ± SEM of the bioluminescence intensities (expressed in cpm/cm^2^) while **S9 and S10 Figs** show the evolution of bioluminescence intensities for each mouse for the thorax and the whole body, respectively. **Fig 4B** shows representative images of bioluminescence acquisitions. For Luc^+^ A549 tumor-bearing mice, neither osimertinib nor bevacizumab, alone or in combination, modified tumor growth (**Fig 4A and 4B upper panels, S9 and S10 Figs upper panels**). For Luc^+^ PC9 tumor-bearing mice, while bevacizumab does not modify tumor growth and osimertinib leads to TGI, the combination of these two therapeutic molecules allows tumor regression (**Fig 4A and 4B middle panels, S9 and S10 Figs middle panels**). Finally, for Luc^+^ H1975 tumor-bearing mice, the administration of osimertinib leads to the TGI and the use bevacizumab alone or in combination with osimertinib does not significantly modify tumor growth (**Fig 4A and 4B lower panels, S9 and S10 Figs lower panels**). Consistent with the fact that the mice were immunodeficient, immune cells were not observed with the anti-B220 antibody in NSCLC tumor-bearing mice (**S11 Fig)**. In mice treated with osimertinib plus bevacizumab, more staining with CD31/PECAM antibody was observed, there were also more tumor foci (**S11 Fig)**. Altogether, these results highlight i) the different tumor responses against the EGFR TKI erlotinib and osimerinib and against bevacizumab for A549, PC9 and H1975 tumors and ii) demonstrate the efficacy of osimertinib *plus* bevacizumab in some NSCLC subpopulation.

### Therapeutic effects of osimertinib plus afatinib in orthotopic models of NSCLC

**Fig 5:**
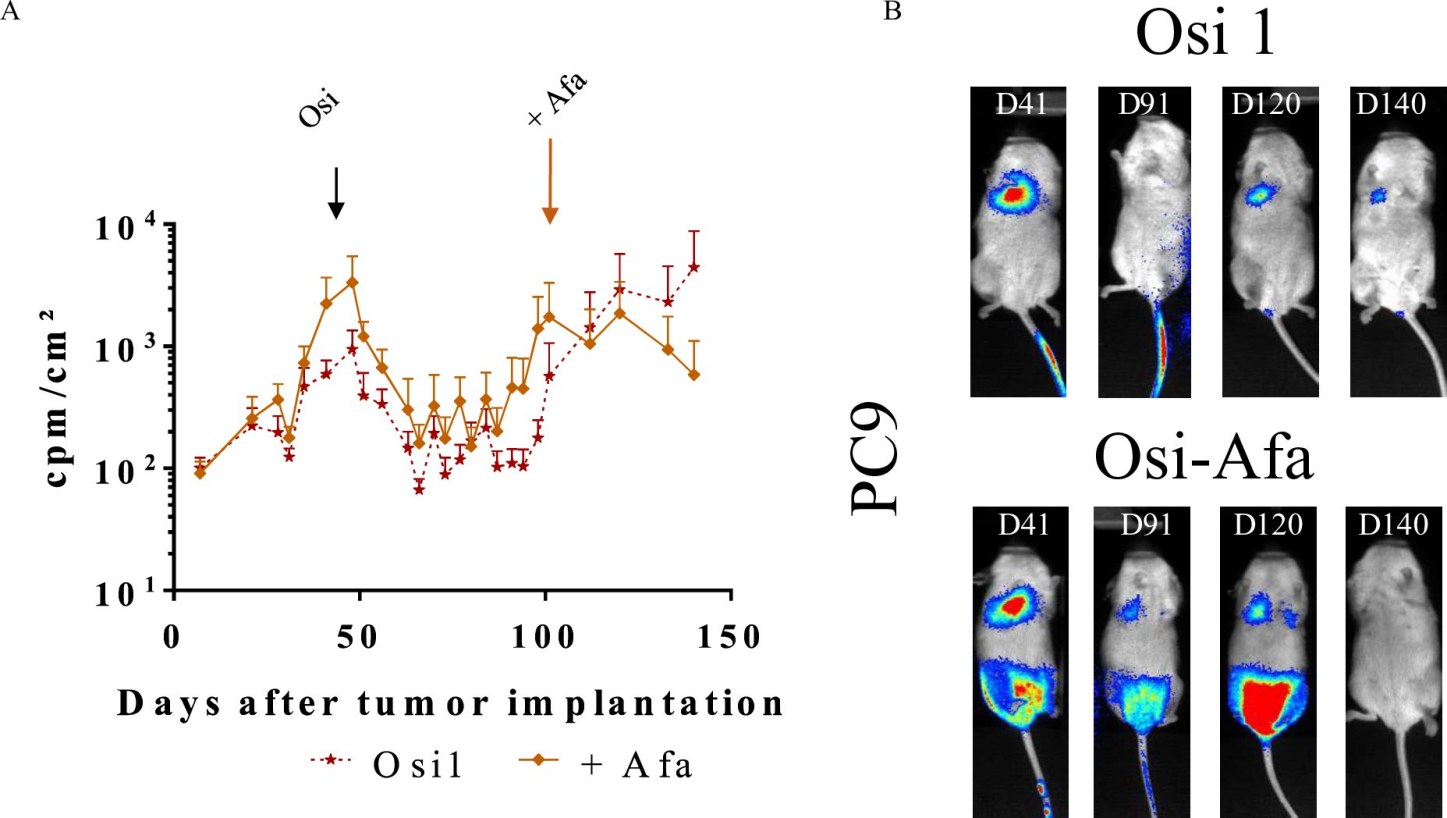
Osimertinib *plus* Afatinib therapeutic effects on tumor growth in orthotopic Luc^+^ NSCLC model in NSG mice. Luc^+^ PC9 tumor bearing mice were treated 5 days a week with osimertinib at 1 mg/kg. When tumor escape occurred, mice were treated additionally, or not (---★---), 2 days a week with afatinib (▬υ▬). Tumor growth was assessed by bioluminescence. **(A)** The results are expressed as cpm/cm^2^ (mean ± SEM, n = 5)**. (B)** Pictures show representative results for bioluminescence of Luc^+^ PC9 tumor bearing mice untreated or treated with osimertinib alone (Osi 1) or with osimertinib *plus* afatinib (Osi-Afa). See S7 Fig for statistical analysis.

Finally, we have assessed whether afatinib, a second generation TKI could be used to treat tumor relapse since adding afatinib to osimertinib could help overcome EGFR-dependent osimertinib resistance, such as secondary C797S mutation or other co-existing EGFR mutations [15]. In order to assess the efficacy of this combination, mice carrying Luc^+^ PC9 tumors were treated with osimertinib at 1 mg/kg daily. When tumor escape was observed, mice were further treated with afatinib at 10 mg/kg daily (**Fig 5 and S8 and S12 Figs).** Results are expressed for the thorax zone (**Fig 5 and S12 Fig upper panel)**, or to the whole body (**S12 Fig lower panel**). **Fig 5A** shows the mean ± SEM of the bioluminescence intensities (express in cpm/cm^2^) while **S12 Fig** shows the evolution of bioluminescence intensities for each mouse. **Fig 5B** shows representative images of bioluminescence acquisitions. When tumor relapse was observed in osimertinib-treated mice, the addition of Afatinib restored TGI. Statistical significance was not yet reached, but the experiment was stopped because one of the mice treated with osi alone had to be euthanized due to the size reached by the tumor. It should be noted that a preliminary experiment was performed with a small number of Luc^+^ H1975 tumor-bearing mice but no therapeutic effect was observed with afatinib combined to osimertinib (**S13 Fig; n = 2).** Interestingly, we also assessed the effect of the MEK 1/2 inhibitor selumetinib (AZD6244, Astrazeneca, Cambridge, UK) and of the HDAC inhibitor simvastatin (**S14 Fig**). In both cases, no therapeutic gain was observed on the primary lung tumor for mice bearing Luc^+^ PC9 tumors (**S14 Fig upper panel**) while a slow effect was obtained when considering the total body (**S14 Fig lower panel**). The expression of p-ERK was not significantly different in mice treated with osimertinib compared with those treated with osimertinib and afatinib in the lung sections examined **(S15 Fig)**.

Together, these results showed that despite the development of resistance to Osimertinib, the EGFR TKI afatinib could represent an opportunity for patients with relapsed NSCLC.

## Discussion

We have established cell lines expressing the luciferase gene from lines having varied genetic backgrounds commonly found in patients with pulmonary adenocarcinoma. These cell lines retain the characteristics of response to genotoxic agents and inhibitors as we showed using a large number of these molecules (Fig 1). The establishment of Luc^+^ lines allows the longitudinal monitoring of cells, tumor formation, response to treatments and dissemination in immunocompromised mice. This methodology makes it possible to limit the number of animals used. These bioluminescent preclinical models allow the detection of a small number of cells present in the body, even in deep organs such as the lung. A 3-dimensional reconstruction of the orthotopic tumor was possible (Fig 2). We have previously shown that these models make it possible to detect and sample foci of disseminated cells in the body, not visible to the naked eye even during autopsy [14].

The four cell lines generated can be implanted subcutaneously to generate xenografts. However, we have focused on generating orthotopic tumors that in response to treatment more realistically mimic what is happening in patients. The implantation of tumors from cells injected into the tail vein is very efficient with an engraftment of practically 100% for 3 lines: Luc^+^ A549, Luc^+^ PC9 and Luc^+^ H1975 cells in NSG mice. Engraftment is rapid with tumors visible by bioluminescence in less than ten days after the injection and reaching an appropriate volume for the start of treatment 20 days after tumor cells injection. In response to EGFR inhibitors, erlotinib and osimertinib, tumors respond to treatment according to the sensitivity conferred by the status of the EGFR gene mutation of each lineage (Fig 3). Interestingly, for both erlotinib and osimertinib, a dose response is observed for each inhibitor administered (Fig 3) whereas in the clinic, these two inhibitors are given at a fixed dose whatever the patient’s weight [16]. The results obtained show the efficacy of these treatments with, for high doses of osimertinib (5 mg/kg), a complete disappearance of the signal for some Luc^+^PC-9 tumours.

Some studies suggest that the combination of chemotherapy with anti-angiogenics and immunotherapy could be a therapeutic option [16]. Treatment of Luc^+^ PC9- and Luc^+^ H1975 tumors with bevacizumab alone does not lead to a significant effect on tumor growth compared to untreated mice. On the other hand, co-treatment of osimertinib and bevacizumab demonstrated a greater TGI than in mice treated with osimertinib alone. These results show the relevance of concomitant treatments targeting angiogenesis with osimertinib, which could increase the efficacy of osimertinib by preventing or at least delay the emergence of resistant tumor clones. However, phase 2 and 3 clinical trials have not shown any efficacy of this combination on PFS [17–19]. This difference between what is observed in mouse and human could be due to the use of immunodeficient mice while using a human monoclonal antibody. Immunohistochemical analysis of endothelial cells using CD31 antibody was not conclusive, although a trend was observed. New experiments analyzing a larger number of tumors and with longitudinal follow-up will be necessary.

Finally, when we tested combinations of osimertinib with afatinib, selumetinib or simvastatin in response to tumor relapsing from osimertinib treatment we showed that only afatinib has a therapeutic benefit. A few studies have demonstrated the benefit of using afatinib when resistance to osimertinib occurs probably through its pan activity on other TK such as HER3. A low dose of osimertinib was used in the studies (1 mg/kg); thus, it cannot be excluded that a modest effect of afatinib could also be related to a fuller inhibition of EGFR. Furthermore, alternation of osimertinib and afatinib has been proposed as a way to overcome osimertinib resistance since each drug has a distinct binding site [20, 21]. We didn’t observe a different expression of p-ERK in mice treated with osimertinib compared with those treated with osimertinib and afatinib. Again, new experiments analyzing a larger number of tumors and with longitudinal follow-up will be necessary. Additional treatment strategies to overcome resistance mechanisms may be tested in these models to better understand how EGFR-TKI-specific resistance mechanisms evolves [22, 23].

In conclusion, we have developed preclinical models of NSCLC with very high engraftment efficiency, easy monitoring of tumor growth, monitoring of cell dissemination in the body and the possibility to recover micro-metastases for analysis. These models are therefore of great interest for the study of the mechanisms of resistance to anticancer drugs. Furthermore, these models will allow to test the effectiveness of a varied number of treatments and combinations in order to focus with greater chances of success on the combinations to be tested in clinical trials.

## Supporting information

S1 Fig Generation and characterization of Luc^+^ NSCLC cell lines. **(A)** Table indicates the vector used for Luc induction and the result of STR assays for the luciferase cell lines in comparison with parental cells. **(B-D)** Wt and Luc^+^ NSCLC cells were treated with different concentrations of etoposide, erlotinib, osimertinib and afatinib and were used for CFU and EC50 assays. **(B)** Representative results of CFU assays. **(C)** The graph shows a representative result used for the EC50 and Min determination. **(D)** The results are expressed as the EC50 (μM) and as the minimal cell viabilities (min; %) obtained with the highest concentration (n = 3 or 4).
(PDF)

S2 Fig Response to inhibitors of Luc^+^ NSCLC cell lines. **(A)** Wt and Luc^+^ NSCLC cells were treated with different concentrations of etoposide (Eto), erlotinib (Erlo), afatinib (Afa) and osimertinib (Osi), and then used for EC50 assays. Results are expressed as the EC50 (n = 6). **(B)** Wt and Luc^+^ NSCLC cells were treated with etoposide, erlotinib, afatinib and osimertinib at the EC50 concentration (EC50), or 10x less (EC50/10) or 10x higher (EC50x10), and then used for CFU assays (n = 6 or more).
(PDF)

S3 Fig Response to drug treatment of Luc^+^ NSCLC cell lines. Wt et Luc^+^ NSCLC cells were treated with different concentrations of doxorubicin (Doxo), cisplatin (Cis), carboplatin (Carbo), fluorouracil (5-FU), crizotinib (Crizo), AZD0156, KU55933, AZD6738, panobinostat (Pano), vorinostat (Vori), belinostat (Belino), droxinostat (Droxi), RGFP966, Mkc-3946, olaparib (Ola) and MK1775 and then were used for EC50 assays. The results are expressed as the EC50 (μM) and as the minimal cell viabilities (min; %) obtained with the highest concentration (n = 3 or 4).
(PDF)

S4 Fig Set up of orthotopic models of NSCLC tumor in mice. Representative video of tumor localization assessed using BioImager 4D module and Atlas 3D reconstruction.
(AVI)

S5 Fig Osimertinib and Erlotinib therapeutic effect on tumor growth in orthotopic Luc^+^ NSCLC models. Tumor bearing mice (A549, PC9 and H1975) were treated 5 days a week with osimertinib at 1 (---★---) or 5 (▬★▬) mg/kg or erlotinib at 12.5 (---▯---), 25 (---▯---) or 50 (▬▯▬) mg/kg or not treated (▬). Tumor growth was assessed by bioluminescence. The results are expressed in cpm/cm^2^ for the thorax area with a graph showing the mean ± SEM curves for each experimental condition and graphs showing the monitoring of each mouse individually for each experimental condition.
(PDF)

S6 Fig Osimertinib and Erlotinib therapeutic effects on tumor growth in orthotopic Luc^+^ NSCLC models. Tumor bearing mice (A549, PC9 and H1975) were treated 5 days a week with Osimertinib at 1 (---★---) or 5 (▬★▬) mg/kg or Erlotinib at 12.5 (---▯---), 25 (---▯---) or 50 (▬▯▬) mg/kg or not treated (▬). Tumor growth was assessed by bioluminescence. **(A)** The results are expressed as cpm/cm^2^ for the **whole body** (mean ± SEM, n = 9)**. (B)** Pictures show representative results for bioluminescence of Luc^+^ PC9 tumor bearing mice untreated or treated with osimertinib 1 (Osi 1) or 5 (Osi 5) mg/kg or erlotinib 12.5 (Erlo 12.5), 25 (Erlo 25) or 50 (Erlo 50) mg/kg. **(C)** Pictures show representative results for bioluminescence of Luc^+^ H1975 tumor bearing mice untreated or treated with osimertinib 1 (Osi 1) or 5 (Osi 5) mg/kg or erlotinib 50 (Erlo 50) mg/kg.
(PDF)

S7 Fig Osimertinib and Erlotinib therapeutic effects on tumor growth in orthotopic Luc^+^ NSCLC models. Tumor bearing mice (A549, PC9 and H1975) mice were treated 5 days a week with osimertinib at 1 (---★---) or 5 (▬★▬) mg/kg or erlotinib at 12.5 (---▯---), 25 (---▯---) or 50 (▬▯▬) mg/kg or not treated (▬). The results are expressed in cpm/cm^2^ for the whole body with a graph showing the mean ± SEM curves for each experimental condition and graphs showing the monitoring of each mouse individually for each experimental condition.
(PDF)

S8 Fig Osimertinib and Erlotinib therapeutic effects on tumor growth in orthotopic Luc^+^ NSCLC models. Statistical analysis of the results expressed in cpm/cm^2^ for figures 3, 4 and 5.
(PDF)

S9 Fig Evaluation of Osimertinib *plus* Bevacizumab therapeutic effects on tumor growth in orthotopic Luc^+^ NSCLC models. Tumor bearing mice (A549, PC9 and H1975) mice were treated 5 days a week with osimertinib at 1mg/kg (---★---), or 2 days a week with bevacizumab (▬▯▬), or both (▬X▬) or not treated (▬). Tumor growth was assessed by bioluminescence. The results are expressed in cpm/cm^2^ for the thorax area with a graph showing the mean ± SEM curves for each experimental condition and graphs showing the monitoring of each mouse individually for each experimental condition.
(PDF)

S10 Fig Osimertinib *plus* Bevacizumab therapeutic effects on tumor growth in orthotopic Luc^+^ NSCLC models. Tumor bearing mice (A549, PC9 and H1975) were treated, or not (▬), 5 days a week with osimertinib at 1 (---★---) mg/kg, or 2 days a week with bevacizumab (▬▯▬), or both (▬X▬). Tumor growth was assessed by bioluminescence. The results are expressed in cpm/cm^2^ for the whole body with a graph showing the mean ± SEM curves for each experimental condition and graphs showing the monitoring of each mouse individually for each experimental condition.
(PDF)

S11 Fig B220 and CD31 expressions in tumors treated with Osimertinib plus Bevacizumab. c of NSCLC tumor-bearing mice treated with Osimertinib alone or Osimertinib plus Bevacizumab were hybridized with B220 and CD31 antibodies to detect immune and endothelial cells.
(PDF)

S12 Fig Osimertinib *plus* Afatinib therapeutic effects on tumor growth in orthotopic Luc^+^ PC9 models. Luc^+^ PC9 tumor-bearing mice were treated 5 days a week with osimertinib at 1 mg/kg. When tumor escape was observed, mice were treated additionally, or not (---★---), 2 days a week with afatinib (▬υ▬). Tumor growth was assessed by bioluminescence. The results are expressed in cpm/cm^2^ for the **thorax area** (up) and **whole body** (down) with a graph showing the mean ± SEM curves for each experimental condition and graphs showing the monitoring of each mouse individually for each experimental condition.
(PDF)

S13 Fig Osimertinib *plus* Afatinib therapeutic effects on tumor growth in orthotopic Luc^+^ H1975 models. Luc^+^ H1975 tumor-bearing mice were treated 5 days a week with osimertinib at 1 mg/kg and afatinib 10mg/kg.
(PDF)

S14 Fig Osimertinib *plus* Selumetinib or Simvastatin therapeutic effects on tumor growth in orthotopic Luc^+^ PC9 models. Luc^+^ PC9 tumor-bearing mice were treated 5 days a week with osimertinib at 1 mg/kg and selumetinib at 50mg/kg or simvastatin at 20mg/kg. Tumor growth was assessed by bioluminescence. The results are expressed in cpm/cm^2^ for the **thorax area** (up) and **whole body** (down) with a graph showing the mean ± SEM curves for each experimental condition and graphs showing the monitoring of each mouse individually for each experimental condition.
(PDF)

S15 Fig p-ERK expression in tumors treated with Osimertinib and Afatinib. Lung sections of NSCLC tumor-bearing mice treated with Osimertinib alone or Osimertinib plus Afatinib were hybridized with the p-ERK antibody.
(PDF)

S1 Table EC50 of Luc^+^ NSCLC cell lines to drug treatment. Recapitulative tables of results of EC50 assays including number of experiments (n), means, standard deviation and standard error of mean.
(PDF)

S2 Table Minimal cell number for EC50 a of Luc^+^ NSCLC cell lines. Recapitulative tables of results of Min for EC50 assays including number of experiments (n), means, standard deviation and standard error of mean.
(PDF)
