# Supplementary material for: Afatinib or Bevacizumab in combination with Osimertinib efficiently control tumor development in orthotopic murine models of non-small lung cancer
Source: PLoS One. 2024 Jun 27;19(6):e0304914. doi: 10.1371/journal.pone.0304914 (PMC11210880; doi:10.1371/journal.pone.0304914)

Jarry et al, Fig supplemental 1

A

	Origine	STR on wt cells	Vector for firefly induction	STR on Luc+ cells
A549	ATCC	Exact Match with A549 from ATCC database (100%)	RediFect Red-FLuc-Puromycin	Exact Match with A549 from ATCC database (100%)
H1975	ATCC	Exact Match with H1975 from ATCC database (100%)	RediFect Red-FLuc-Puromycin	Match with H1975 from ATCC database (93%)
PC9	Sigma-Aldrich	Match with PC-9 from DSMZ STR database (94%)	pGL4.51[luc2/CMV/Neo]	Match with PC-9 from DSMZ STR database (94%)
HCC827	ATCC	Exact Match with HCC827 from ATCC database (100%)	pGL4.51[luc2/CMV/Neo]	Exact Match with HCC827 from ATCC database (100%)

B

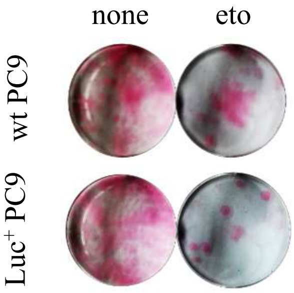

C

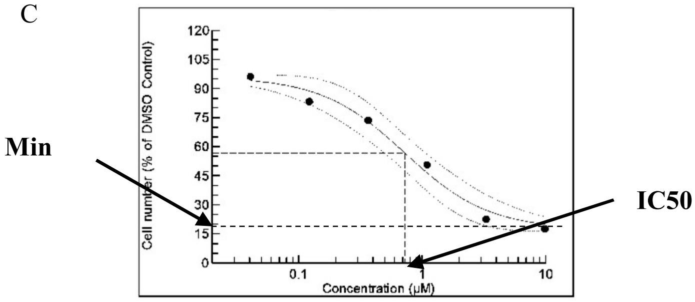

D

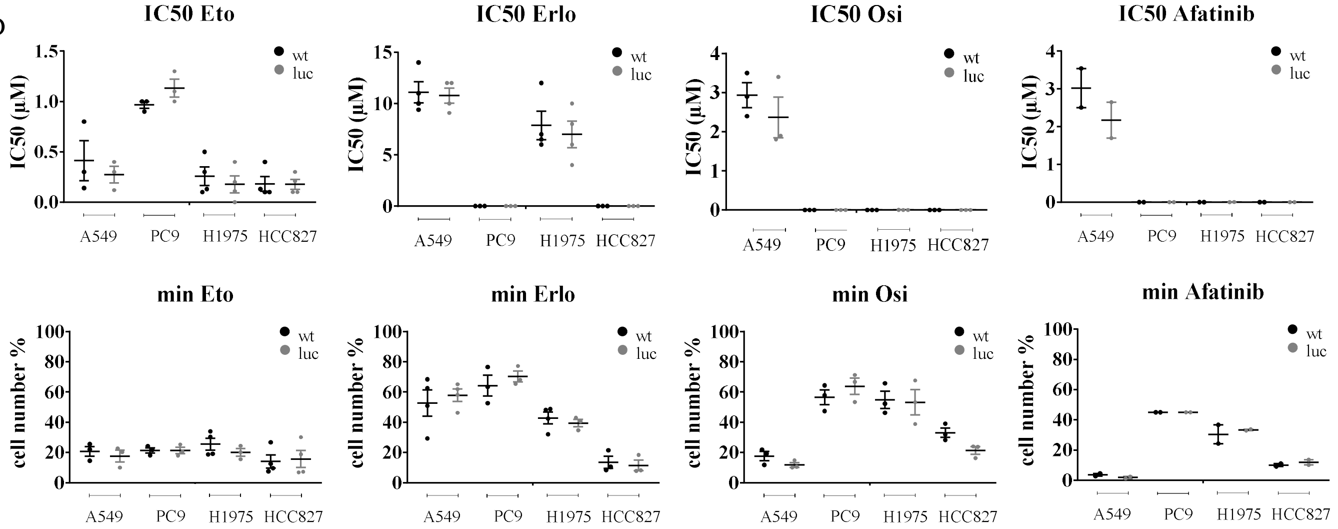

Supplement: S1 Fig — (A) Table indicates the vector used for Luc induction and the result of STR assays for the luciferase cell lines in comparison with parental cells. (B-D) Wt and Luc+ NSCLC cells were treated with different concentrations of etoposide, erlotinib, osimertinib and afatinib and were used for CFU and EC50 assays. (B) Representative results of CFU assays. (C) The graph shows a representative result used for the EC50 and Min determination. (D) The results are expressed as the EC50 (μM) and as the minimal cell viabilities (min; %) obtained with the highest concentration (n = 3 or 4). (PDF) [file pone.0304914.s001.pdf]
