# Supplementary figures and images for: Afatinib or Bevacizumab in combination with Osimertinib efficiently control tumor development in orthotopic murine models of non-small lung cancer
Source: PLoS One. 2024 Jun 27;19(6):e0304914. doi: 10.1371/journal.pone.0304914 (PMC11210880; doi:10.1371/journal.pone.0304914)

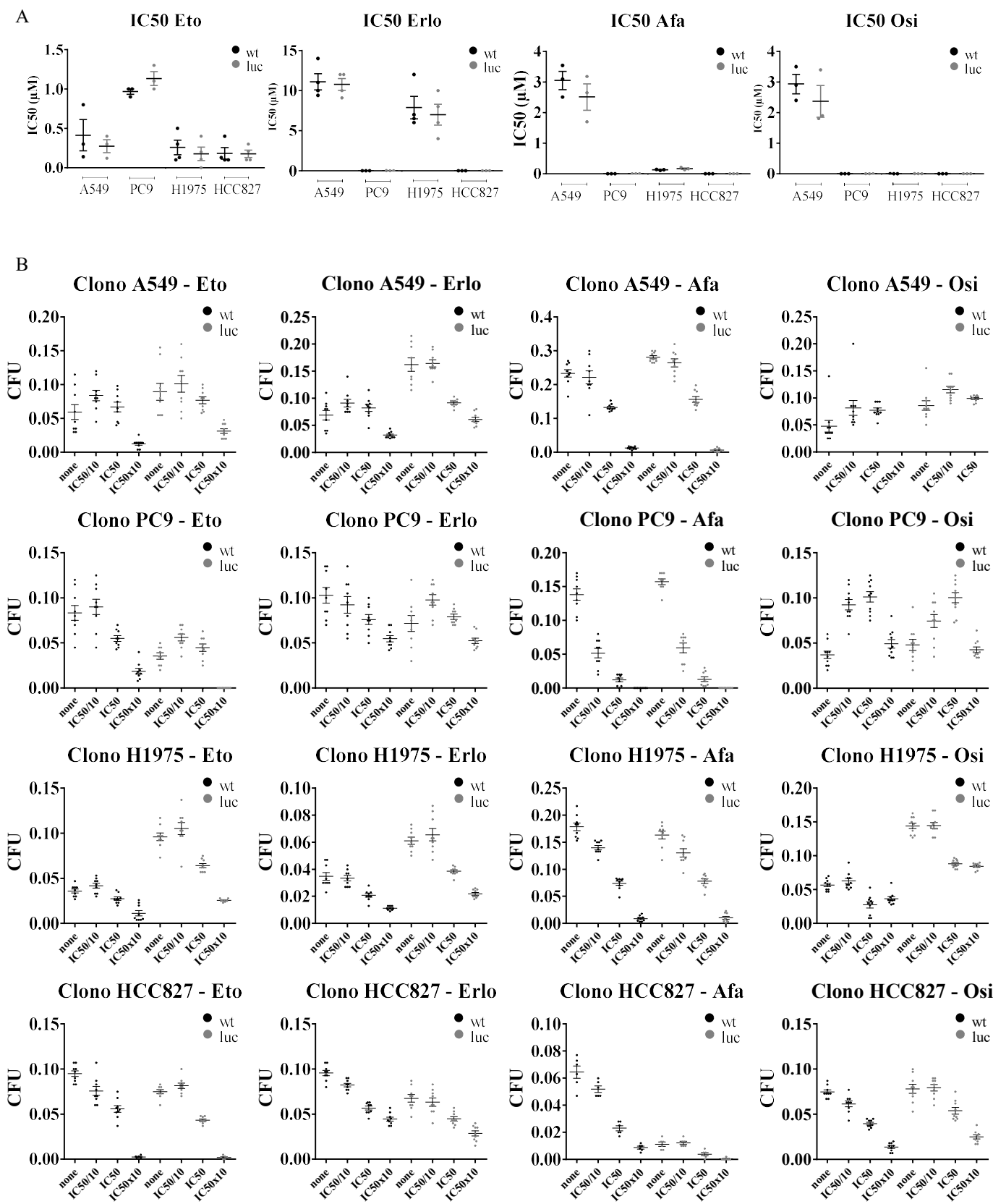

Supplement: S2 Fig — (A) Wt and Luc+ NSCLC cells were treated with different concentrations of etoposide (Eto), erlotinib (Erlo), afatinib (Afa) and osimertinib (Osi), and then used for EC50 assays. Results are expressed as the EC50 (n = 6). (B) Wt and Luc+ NSCLC cells were treated with etoposide, erlotinib, afatinib and osimertinib at the EC50 concentration (EC50), or 10x less (EC50/10) or 10x higher (EC50x10), and then used for CFU assays (n = 6 or more). (PDF) [file pone.0304914.s002.pdf]

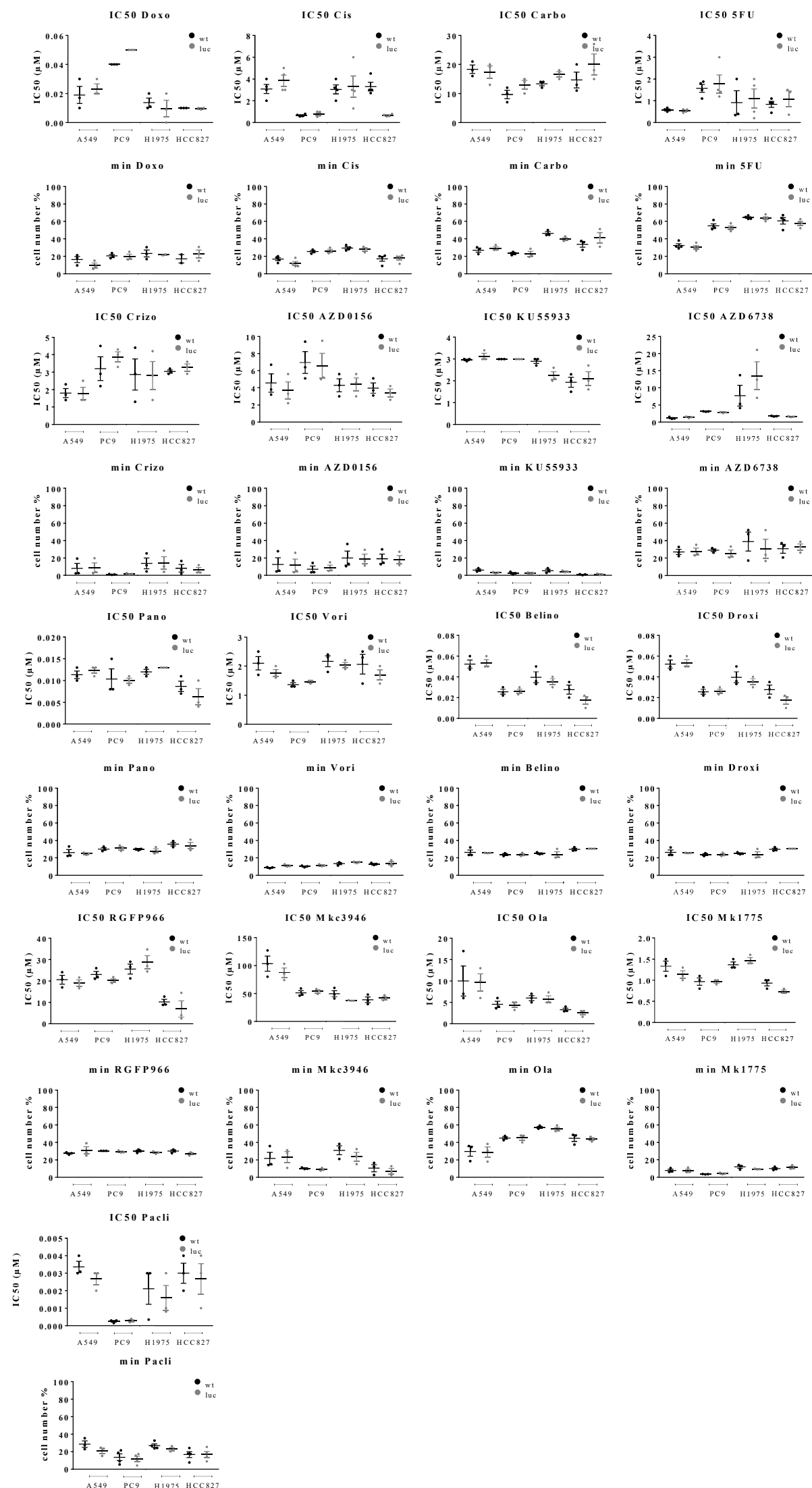

Supplement: S3 Fig — Wt et Luc+ NSCLC cells were treated with different concentrations of doxorubicin (Doxo), cisplatin (Cis), carboplatin (Carbo), fluorouracil (5-FU), crizotinib (Crizo), AZD0156, KU55933, AZD6738, panobinostat (Pano), vorinostat (Vori), belinostat (Belino), droxinostat (Droxi), RGFP966, Mkc-3946, olaparib (Ola) and MK1775 and then were used for EC50 assays. The results are expressed as the EC50 (μM) and as the minimal cell viabilities (min; %) obtained with the highest concentration (n = 3 or 4). (PDF) [file pone.0304914.s003.pdf]

A549

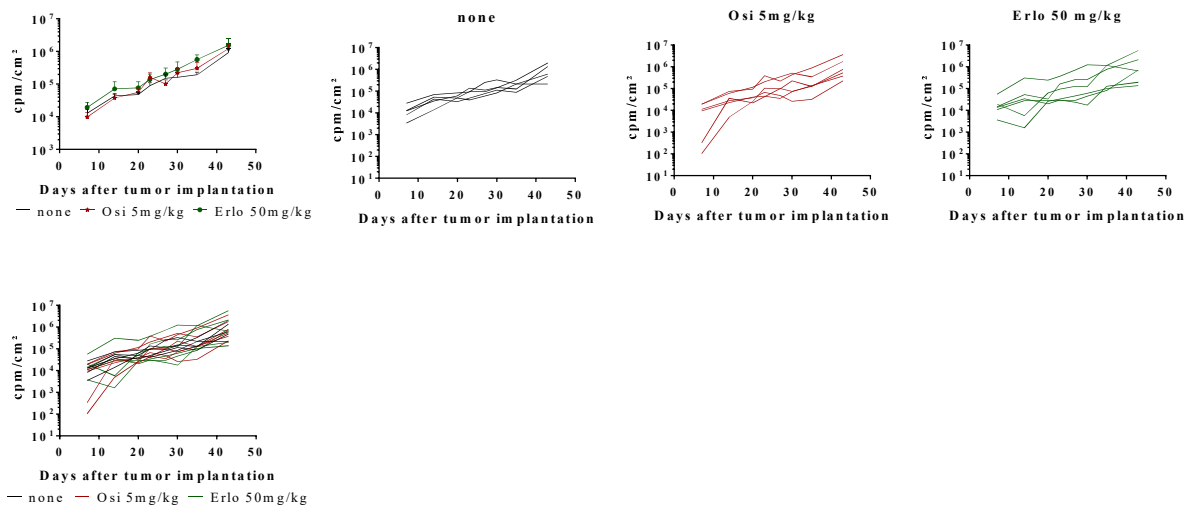

PC9

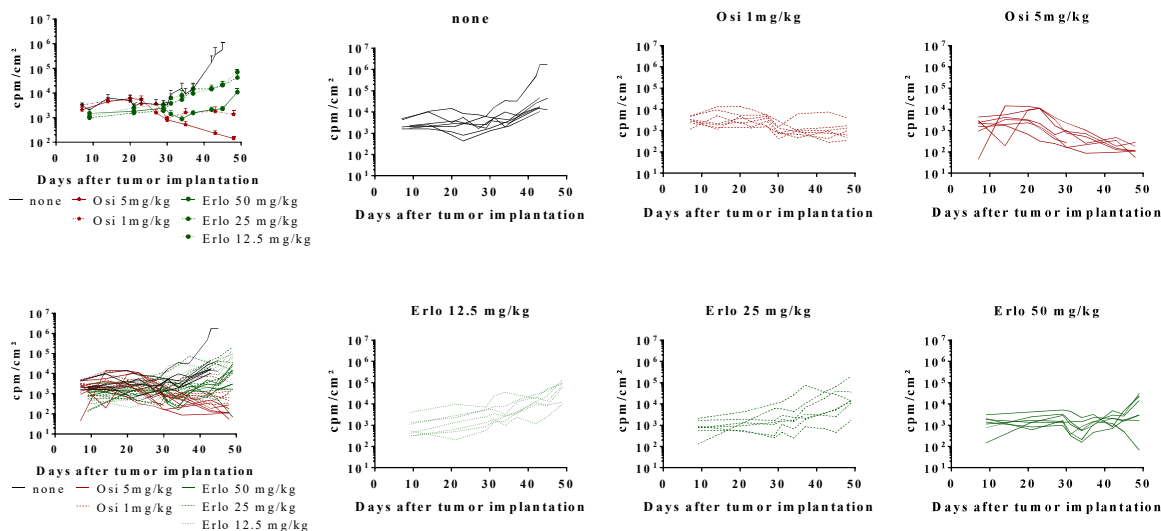

H1975

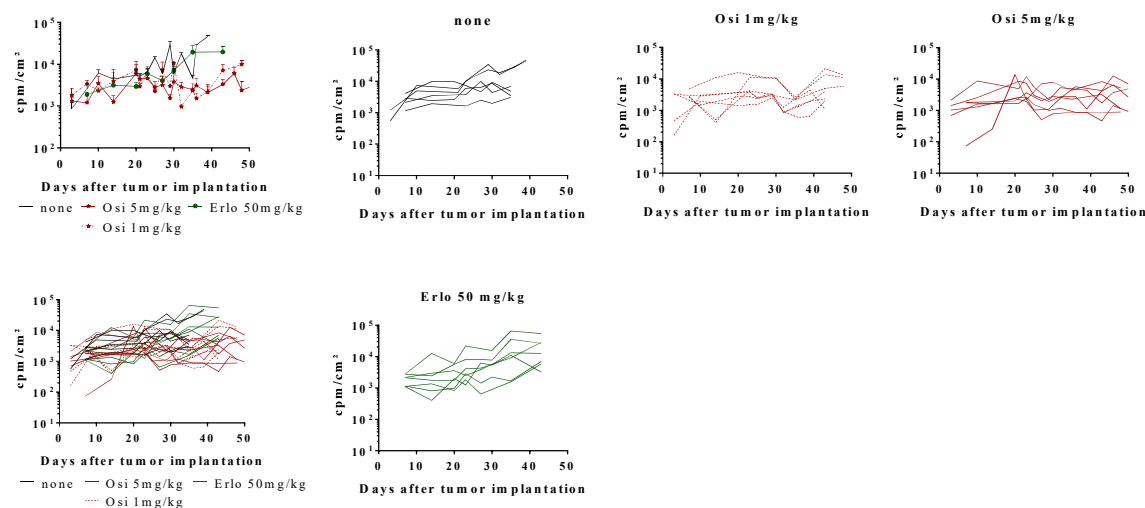

Supplement: S5 Fig — Tumor bearing mice (A549, PC9 and H1975) were treated 5 days a week with osimertinib at 1 (---★---) or 5 (▬★▬) mg/kg or erlotinib at 12.5 (---▯---), 25 (---▯---) or 50 (▬▯▬) mg/kg or not treated (▬). Tumor growth was assessed by bioluminescence. The results are expressed in cpm/cm2 for the thorax area with a graph showing the mean ± SEM curves for each experimental condition and graphs showing the monitoring of each mouse individually for each experimental condition. (PDF) [file pone.0304914.s005.pdf]

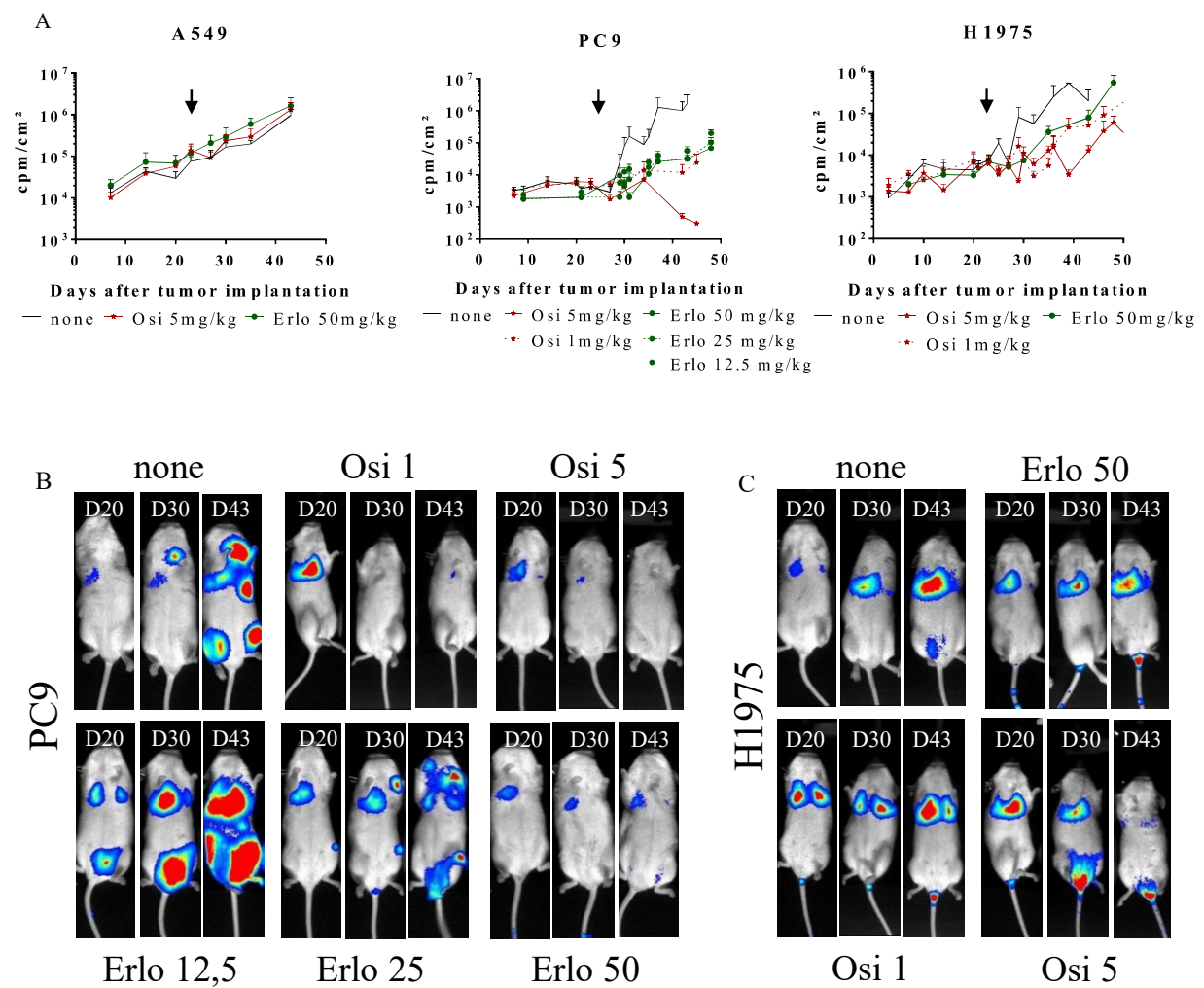

Supplement: S6 Fig — Tumor bearing mice (A549, PC9 and H1975) were treated 5 days a week with Osimertinib at 1 (---★---) or 5 (▬★▬) mg/kg or Erlotinib at 12.5 (---▯---), 25 (---▯---) or 50 (▬▯▬) mg/kg or not treated (▬). Tumor growth was assessed by bioluminescence. (A) The results are expressed as cpm/cm2 for the whole body (mean ± SEM, n = 9). (B) Pictures show representative results for bioluminescence of Luc+ PC9 tumor bearing mice untreated or treated with osimertinib 1 (Osi 1) or 5 (Osi 5) mg/kg or erlotinib 12.5 (Erlo 12.5), 25 (Erlo 25) or 50 (Erlo 50) mg/kg. (C) Pictures show representative results for bioluminescence of Luc+ H1975 tumor bearing mice untreated or treated with osimertinib 1 (Osi 1) or 5 (Osi 5) mg/kg or erlotinib 50 (Erlo 50) mg/kg. (PDF) [file pone.0304914.s006.pdf]

A549

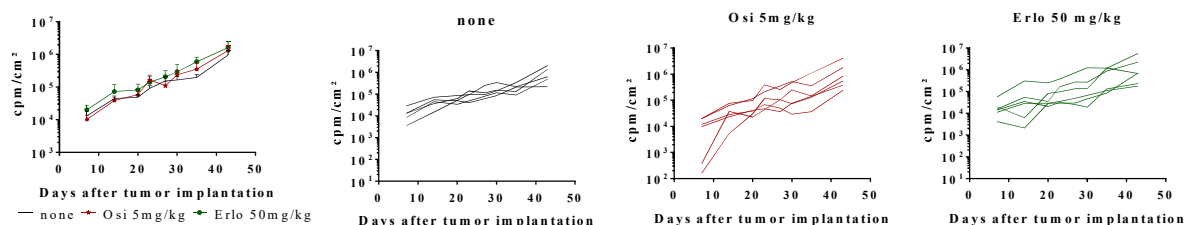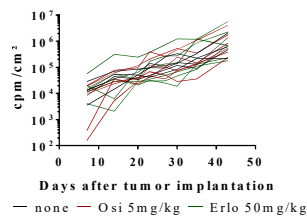

PC9

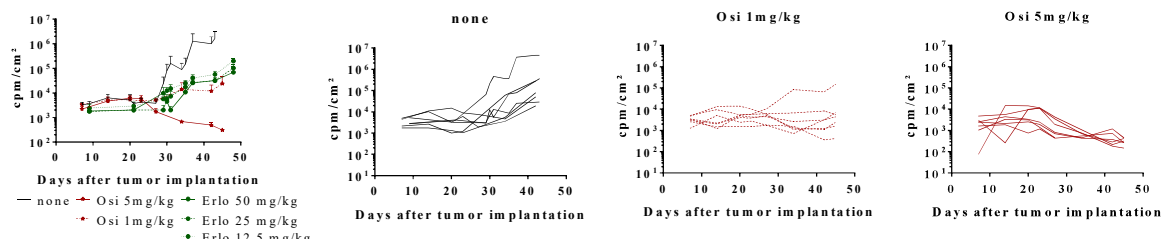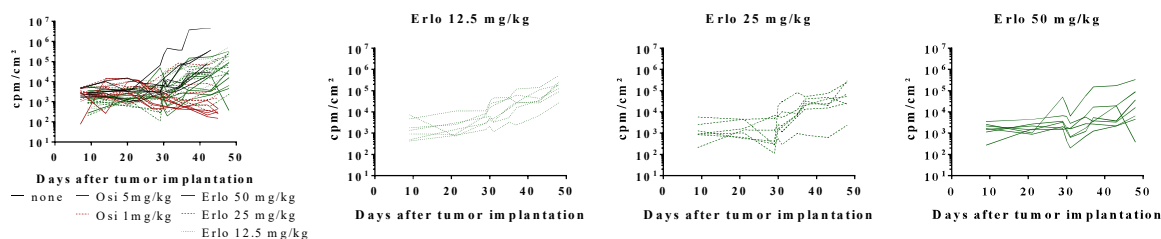

H1975

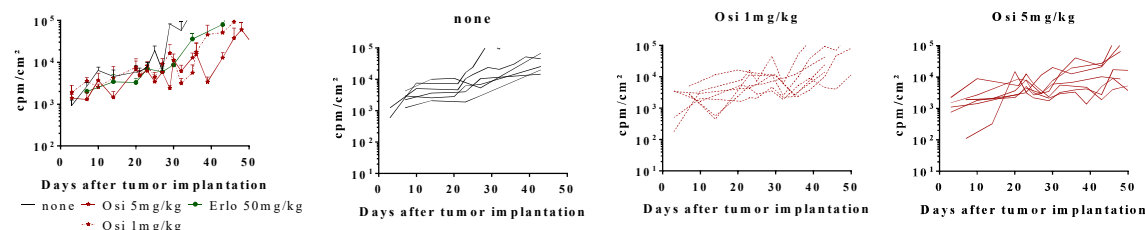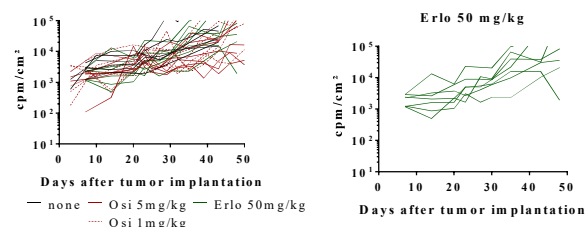

Supplement: S7 Fig — Tumor bearing mice (A549, PC9 and H1975) mice were treated 5 days a week with osimertinib at 1 (---★---) or 5 (▬★▬) mg/kg or erlotinib at 12.5 (---▯---), 25 (---▯---) or 50 (▬▯▬) mg/kg or not treated (▬). The results are expressed in cpm/cm2 for the whole body with a graph showing the mean ± SEM curves for each experimental condition and graphs showing the monitoring of each mouse individually for each experimental condition. (PDF) [file pone.0304914.s007.pdf]

A549

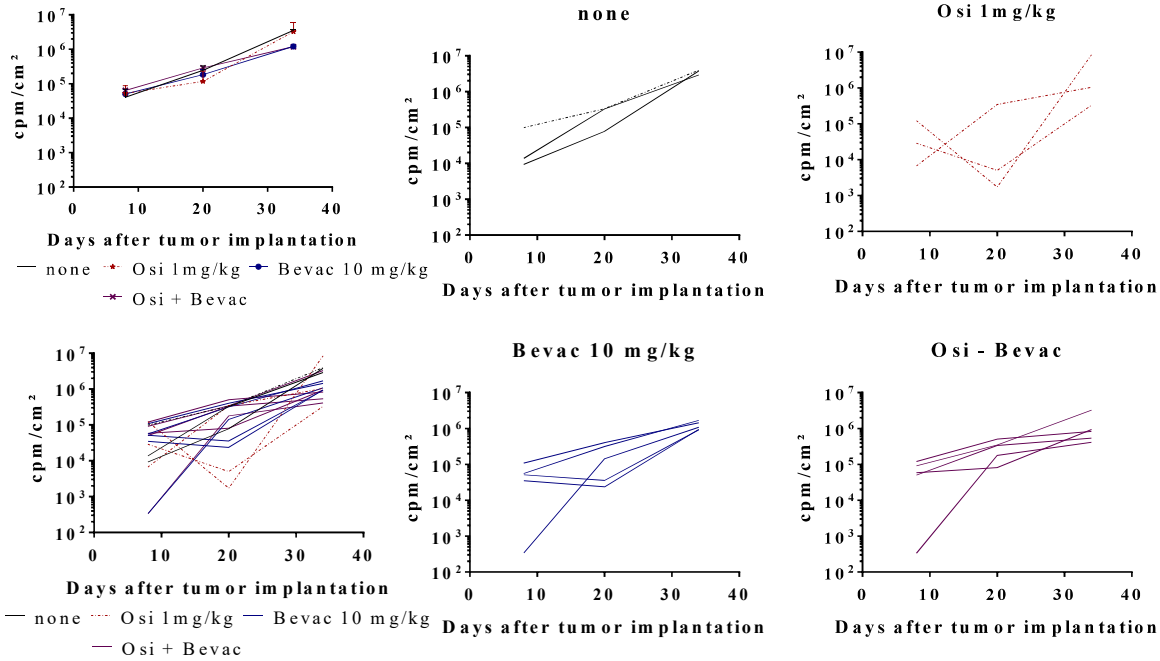

PC9

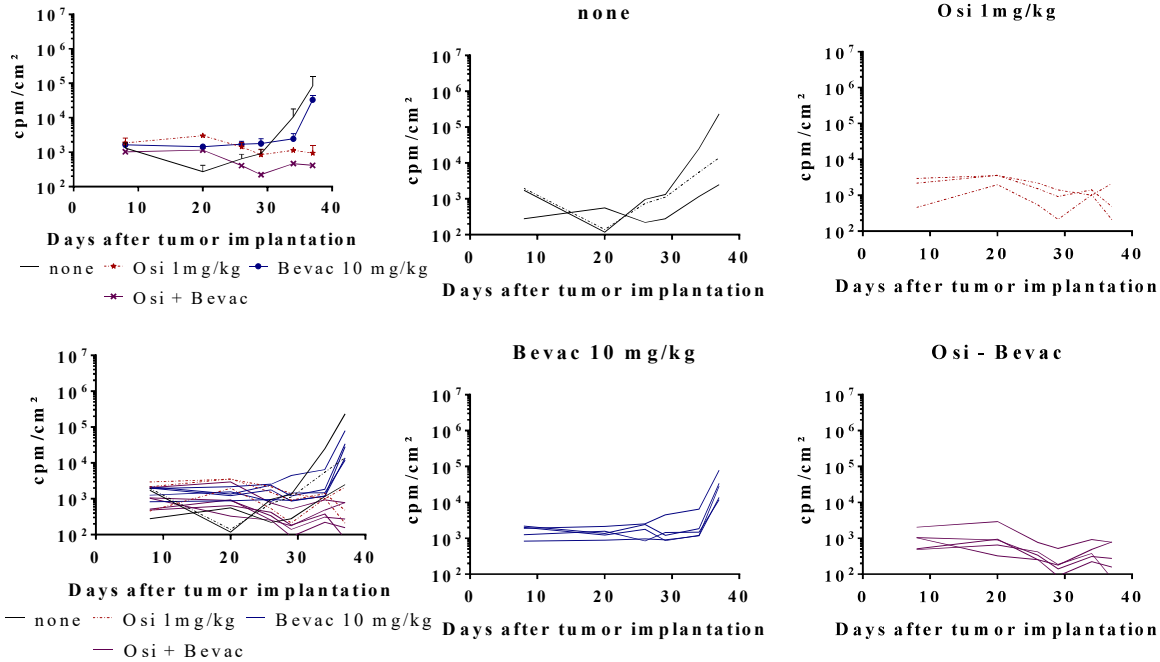

H1975

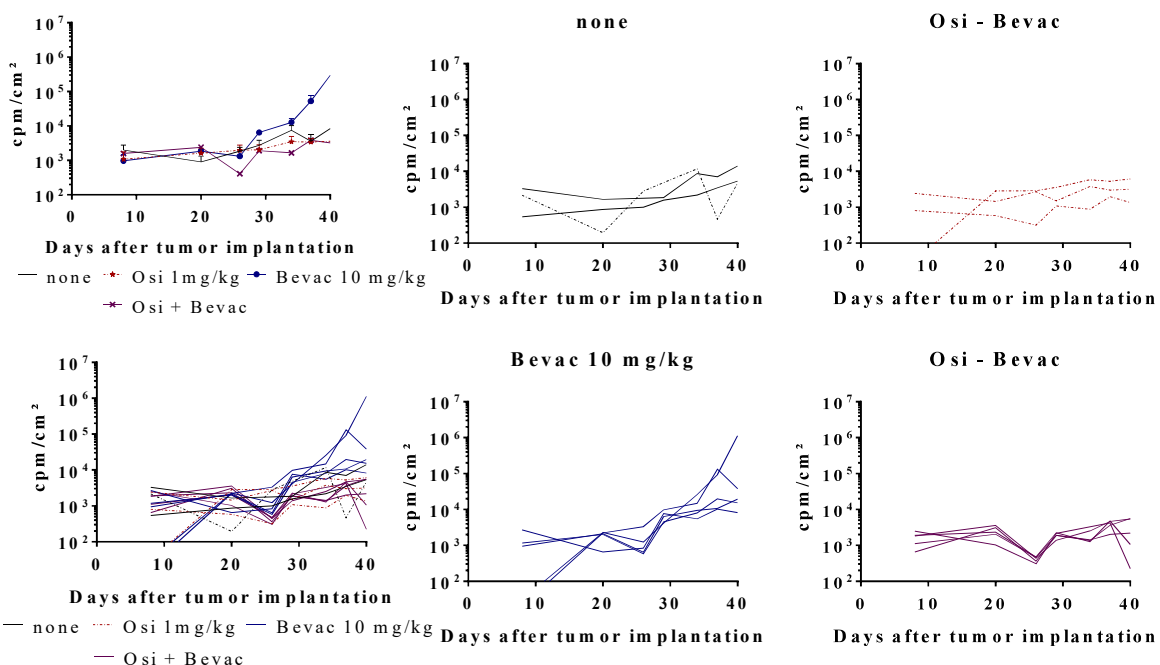

Supplement: S9 Fig — Tumor bearing mice (A549, PC9 and H1975) mice were treated 5 days a week with osimertinib at 1mg/kg (---★---), or 2 days a week with bevacizumab (▬▯▬), or both (▬X▬) or not treated (▬). Tumor growth was assessed by bioluminescence. The results are expressed in cpm/cm2 for the thorax area with a graph showing the mean ± SEM curves for each experimental condition and graphs showing the monitoring of each mouse individually for each experimental condition. (PDF) [file pone.0304914.s009.pdf]

A549

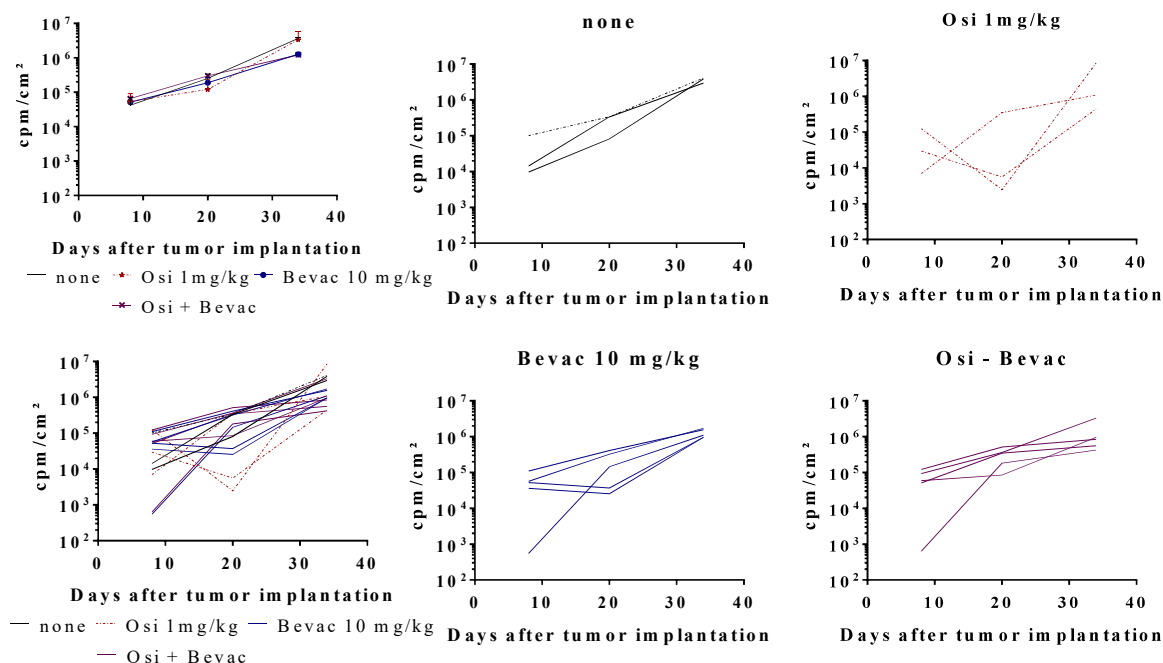

PC9

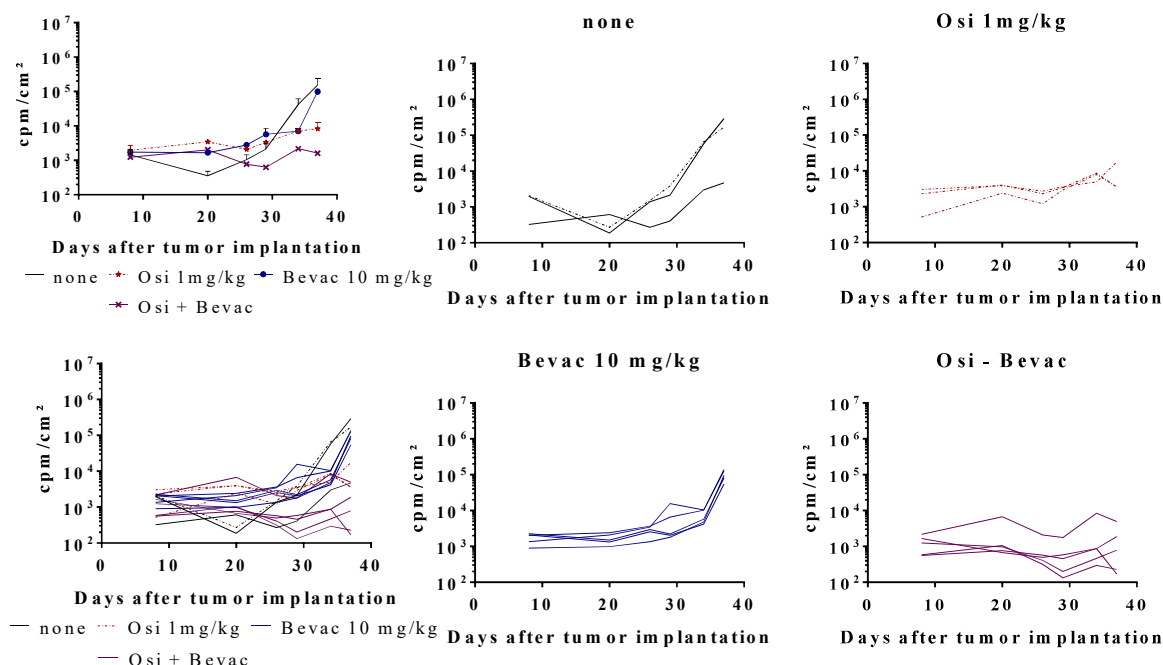

H1975

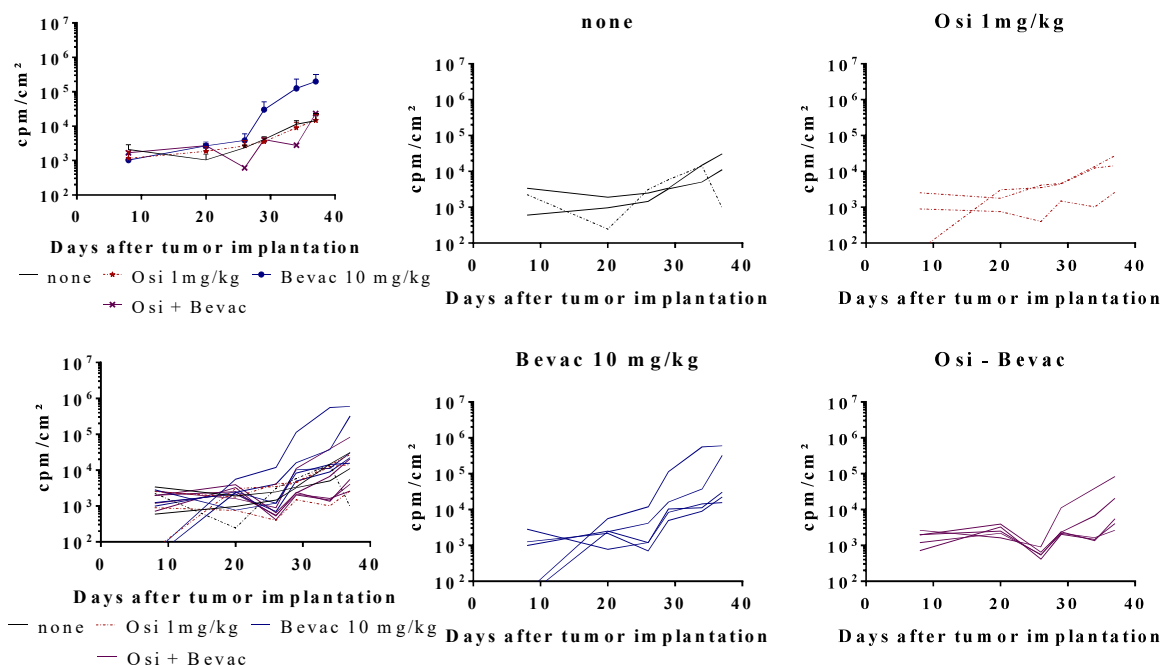

Supplement: S10 Fig — Tumor bearing mice (A549, PC9 and H1975) were treated, or not (▬), 5 days a week with osimertinib at 1 (---★---) mg/kg, or 2 days a week with bevacizumab (▬▯▬), or both (▬X▬). Tumor growth was assessed by bioluminescence. The results are expressed in cpm/cm2 for the whole body with a graph showing the mean ± SEM curves for each experimental condition and graphs showing the monitoring of each mouse individually for each experimental condition. (PDF) [file pone.0304914.s010.pdf]

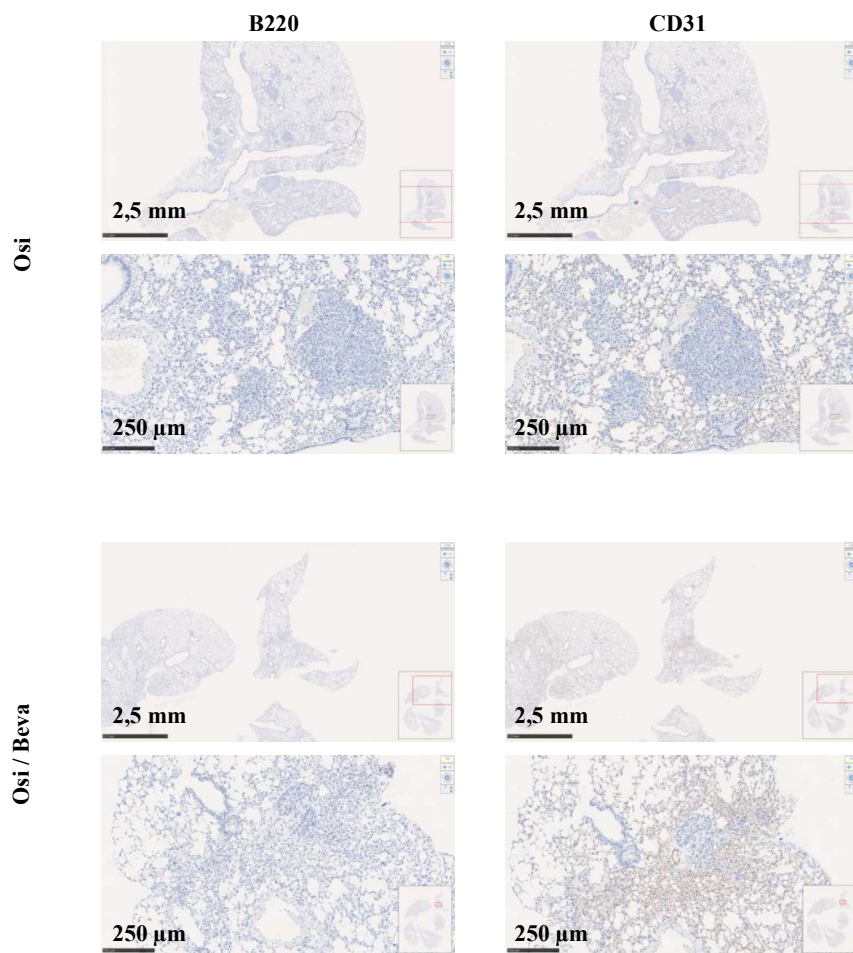

Supplement: S11 Fig — c of NSCLC tumor-bearing mice treated with Osimertinib alone or Osimertinib plus Bevacizumab were hybridized with B220 and CD31 antibodies to detect immune and endothelial cells. (PDF) [file pone.0304914.s011.pdf]

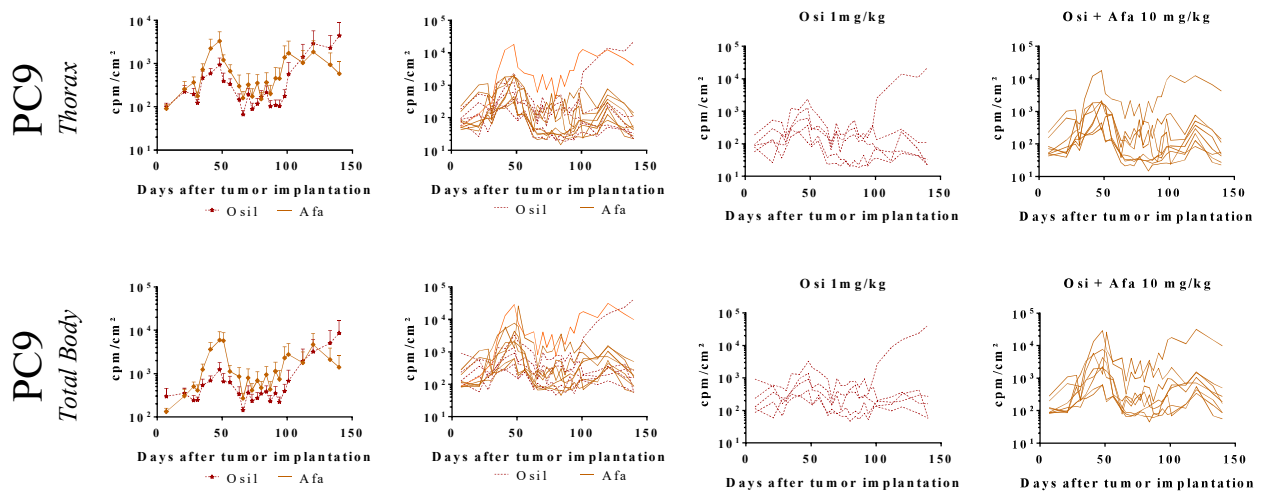

Supplement: S12 Fig — Luc+ PC9 tumor-bearing mice were treated 5 days a week with osimertinib at 1 mg/kg. When tumor escape was observed, mice were treated additionally, or not (---★---), 2 days a week with afatinib (▬υ▬). Tumor growth was assessed by bioluminescence. The results are expressed in cpm/cm2 for the thorax area (up) and whole body (down) with a graph showing the mean ± SEM curves for each experimental condition and graphs showing the monitoring of each mouse individually for each experimental condition. (PDF) [file pone.0304914.s012.pdf]

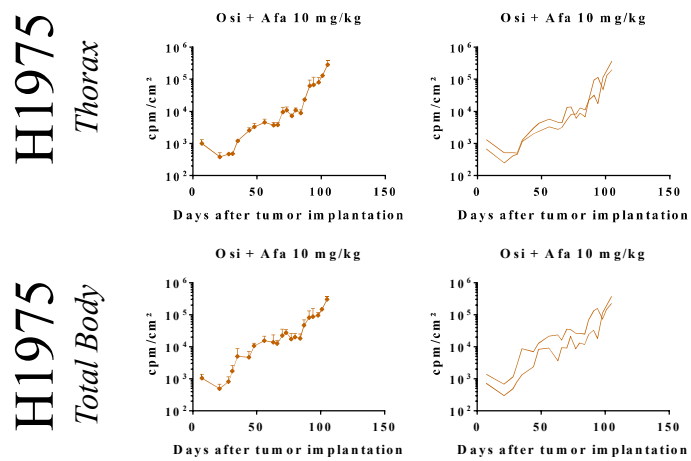

Supplement: S13 Fig — Luc+ H1975 tumor-bearing mice were treated 5 days a week with osimertinib at 1 mg/kg and afatinib 10mg/kg. (PDF) [file pone.0304914.s013.pdf]

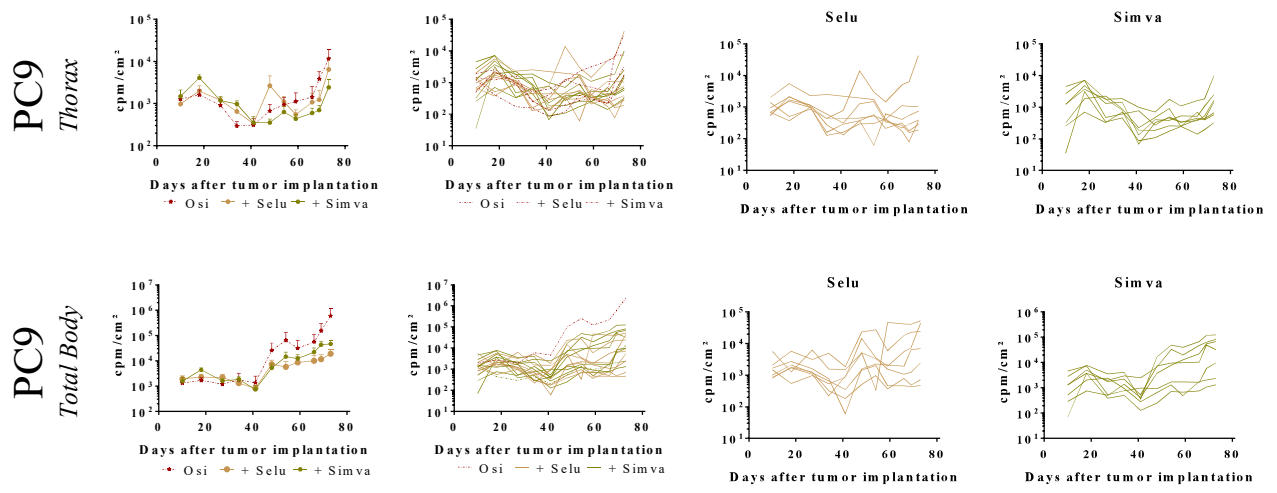

Supplement: S14 Fig — Luc+ PC9 tumor-bearing mice were treated 5 days a week with osimertinib at 1 mg/kg and selumetinib at 50mg/kg or simvastatin at 20mg/kg. Tumor growth was assessed by bioluminescence. The results are expressed in cpm/cm2 for the thorax area (up) and whole body (down) with a graph showing the mean ± SEM curves for each experimental condition and graphs showing the monitoring of each mouse individually for each experimental condition. (PDF) [file pone.0304914.s014.pdf]

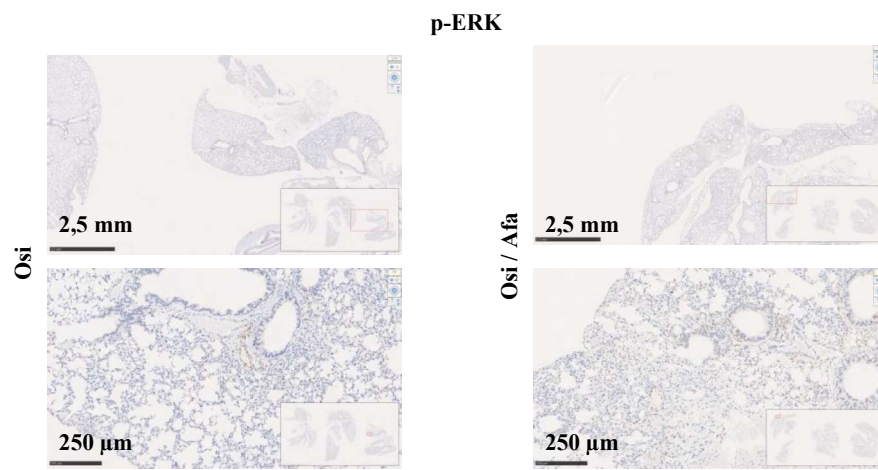

Supplement: S15 Fig — Lung sections of NSCLC tumor-bearing mice treated with Osimertinib alone or Osimertinib plus Afatinib were hybridized with the p-ERK antibody. (PDF) [file pone.0304914.s015.pdf]
