# Supplementary material for: Afatinib or Bevacizumab in combination with Osimertinib efficiently control tumor development in orthotopic murine models of non-small lung cancer
Source: PLoS One. 2024 Jun 27;19(6):e0304914. doi: 10.1371/journal.pone.0304914 (PMC11210880; doi:10.1371/journal.pone.0304914)
Supplement: S8 Fig — Statistical analysis of the results expressed in cpm/cm2 for figures 3, 4 and 5. (PDF) [file pone.0304914.s008.pdf]

Fig3 Lung

A549

None	vs	Osi 5 mg/kg
F test to compare variances		
F, DFn, Dfd	1,749, 7, 7	
P value	0,4781	
P value summary	ns	
Significantly different (P < 0.05)?	No	

None	vs	Erlo 50 mg/kg
F test to compare variances		
F, DFn, Dfd	2,896, 7, 7	
P value	0,184	
P value summary	ns	
Significantly different (P < 0.05)?	No	

PC9

None	vs	Osi 5 mg/kg
F test to compare variances		
F, DFn, Dfd	4018, 15, 8	
P value	<0,0001	
P value summary	****	
Significantly different (P < 0.05)?	Yes	

None	vs	Osi 1 mg/kg
F test to compare variances		
F, DFn, Dfd	12411, 15, 8	
P value	<0,0001	
P value summary	****	
Significantly different (P < 0.05)?	Yes	

None	vs	Erlo 50 mg/kg
F test to compare variances		
F, DFn, Dfd	2906, 15, 8	
P value	<0,0001	
P value summary	****	
Significantly different (P < 0.05)?	Yes	

None	vs	Erlo 25 mg/kg
F test to compare variances		
F, DFn, Dfd	150,8, 15, 8	
P value	<0,0001	
P value summary	****	
Significantly different (P < 0.05)?	Yes	

None	vs	Erlo 12,5 mg/kg
F test to compare variances		
F, DFn, Dfd	57,57, 15, 8	
P value	<0,0001	
P value summary	****	
Significantly different (P < 0.05)?	Yes	

Osi 1 mg/kg	vs	Osi 5 mg/kg
F test to compare variances		
F, DFn, Dfd	3,089, 8, 8	
P value	0,1313	
P value summary	ns	
Significantly different (P < 0.05)?	No	

H1975

None	vs	Osi 5 mg/kg
F test to compare variances		
F, DFn, Dfd	12,57, 15, 20	
P value	<0,0001	
P value summary	****	
Significantly different (P < 0.05)?	Yes	

None	vs	Osi 1 mg/kg
F test to compare variances		
F, DFn, Dfd	14,62, 15, 19	
P value	<0,0001	
P value summary	****	
Significantly different (P < 0.05)?	Yes	

None	vs	Erlo 50 mg/kg
F test to compare variances		
F, DFn, Dfd	4,355, 8, 15	
P value	0,0138	
P value summary	*	
Significantly different (P < 0.05)?	Yes	

Erlo 50 mg/kg	vs	Erlo 25 mg/kg
F test to compare variances		
F, DFn, Dfd	19,26, 8, 8	
P value	0,0004	
P value summary	***	
Significantly different (P < 0.05)?	Yes	
Erlo 50 mg/kg	vs	Erlo 12,5 mg/kg
F test to compare variances		
F, DFn, Dfd	50,48, 8, 8	
P value	<0,0001	
P value summary	****	
Significantly different (P < 0.05)?	Yes	
Erlo 25 mg/kg	vs	Erlo 12,5 mg/kg
F test to compare variances		
F, DFn, Dfd	2,62, 8, 8	
P value	0,1946	
P value summary	ns	
Significantly different (P < 0.05)?	No	

Fig3 Whole Body

A549

None	vs	Osi 5 mg/kg
F test to compare variances		
F, DFn, Dfd	1,87, 7, 7	
P value	0,4277	
P value summary	ns	
Significantly different (P < 0.05)?	No	
None	vs	Erlo 50 mg/kg
F test to compare variances		
F, DFn, Dfd	2,937, 7, 7	
P value	0,1786	
P value summary	ns	
Significantly different (P < 0.05)?	No	

PC9

None	vs	Osi 5 mg/kg
F test to compare variances		
F, DFn, Dfd	40527, 14, 7	
P value	<0,0001	
P value summary	****	
Significantly different (P < 0.05)?	Yes	
None	vs	Osi 1 mg/kg
F test to compare variances		
F, DFn, Dfd	5694, 14, 7	
P value	<0,0001	
P value summary	****	
Significantly different (P < 0.05)?	Yes	
None	vs	Erlo 50 mg/kg
F test to compare variances		
F, DFn, Dfd	608,3, 14, 8	
P value	<0,0001	

H1975

None	vs	Osi 5 mg/kg
F test to compare variances		
F, DFn, Dfd	5,303, 13, 20	
P value	0,0009	
P value summary	***	
Significantly different (P < 0.05)?	Yes	
None	vs	Osi 1 mg/kg
F test to compare variances		
F, DFn, Dfd	4,084, 18, 13	
P value	0,0131	
P value summary	*	
Significantly different (P < 0.05)?	Yes	
None	vs	Erlo 50 mg/kg
F test to compare variances		
F, DFn, Dfd	1,387, 8, 13	
P value	0,5762	

P value summary	****		P value summary	ns
Significantly different (P < 0.05)?	Yes		Significantly different (P < 0.05)?	No

None	vs	Erlo 25 mg/kg
F test to compare variances		
F, DFn, Dfd	291,9, 14, 8	
P value	<0,0001	
P value summary	****	
Significantly different (P < 0.05)?	Yes	

None	vs	Erlo 12,5 mg/kg
F test to compare variances		
F, DFn, Dfd	75,62, 14, 8	
P value	<0,0001	
P value summary	****	
Significantly different (P < 0.05)?	Yes	

Osi 1 mg/kg	vs	Osi 5 mg/kg
F test to compare variances		
F, DFn, Dfd	7,117, 7, 7	
P value	0,019	
P value summary	*	
Significantly different (P < 0.05)?	Yes	

Erlo 50 mg/kg	vs	Erlo 25 mg/kg
F test to compare variances		
F, DFn, Dfd	2,084, 8, 8	
P value	0,3192	
P value summary	ns	
Significantly different (P < 0.05)?	No	

Erlo 50 mg/kg	vs	Erlo 12,5 mg/kg
F test to compare variances		
F, DFn, Dfd	8,045, 8, 8	
P value	0,0079	
P value summary	**	
Significantly different (P < 0.05)?	Yes	

Erlo 25 mg/kg	vs	Erlo 12,5 mg/kg
F test to compare variances		
F, DFn, Dfd	3,86, 8, 8	
P value	0,0735	
P value summary	ns	
Significantly different (P < 0.05)?	No	

Fig 4 A549

None vs Osi

F test to compare variances	
F, DFn, Dfd	1,146, 2, 2
P value	0,9318
P value summary	ns
Significantly different (P < 0.0	No

None vs Beva

F test to compare variances	
F, DFn, Dfd	9,584, 2, 2
P value	0,189
P value summary	ns
Significantly different (P < 0.0	No

None vs Osi/Beva

F test to compare variances	
F, DFn, Dfd	10,84, 2, 2
P value	0,1689
P value summary	ns
Significantly different (P < 0.0	No

Fig 4 PC9

None vs Osi

F test to compare variances	
F, DFn, Dfd	1632, 5, 5
P value	<0,0001
P value summary	****
Significantly different (P < 0.0	Yes

None vs Beva

F test to compare variances	
F, DFn, Dfd	6,537, 5, 5
P value	0,06
P value summary	ns
Significantly different (P < 0.0	No

None vs Osi/Beva

F test to compare variances	
F, DFn, Dfd	7635, 5, 5
P value	<0,0001
P value summary	****
Significantly different (P < 0.0	Yes

Osi + Beva vs. Osi			Osi + Beva vs. Beva	
Column D	Osi + Bevac		Column D	Osi + Bevac
vs.	vs,		vs.	vs,
Column B	Osi 1mg/kg		Column C	Bevac 10 mg/kg
Unpaired t test			Unpaired t test	
P value	0,0311		P value	0,2488
P value summary	*		P value summary	ns
Significantly different (P < 0.05)?	Yes		Significantly different (P < 0.05)?	No
One- or two-tailed P value?	Two-tailed		One- or two-tailed P value?	Two-tailed
t, df	t=2,507 df=10		t, df	t=1,225 df=10
How big is the difference?			How big is the difference?	
Mean ± SEM of column B	1538 ± 334, n=6		Mean ± SEM of column C	7081 ± 5278, n=6
Mean ± SEM of column D	615,3 ± 154,4, n=6		Mean ± SEM of column D	615,3 ± 154,4, n=6
Difference between means	-922,6 ± 368		Difference between means	-6466 ± 5280
95% confidence interval	-1742 to -102,7		95% confidence interval	-18230 to 5299
R squared (eta squared)	0,386		R squared (eta squared)	0,1304

F test to compare variances			F test to compare variances	
F, DFn, Dfd	4,677, 5, 5		F, DFn, Dfd	1168, 5, 5
P value	0,1157		P value	<0,0001
P value summary	ns		P value summary	****
Significantly different (P < 0.05)?	No		Significantly different (P < 0.05)?	Yes

Fig 4 H1975

None vs Osi

F test to compare variances	
F, DFn, Dfd	1,02, 12, 6
P value	>0,9999
P value summary	ns
Significantly different (P < 0.0	No

None vs Beva

F test to compare variances	
F, DFn, Dfd	2741, 6, 6
P value	<0,0001
P value summary	****
Significantly different (P < 0.0	Yes

None vs Osi/Beva

F test to compare variances	
F, DFn, Dfd	1,089, 9, 6
P value	0,9525
P value summary	ns
Significantly different (P < 0.0	No

Fig 5 - Osi +/- Afa

Table Analyzed	Data 1
Column B	Osi-Afa
vs.	vs,
Column A	Afa
Unpaired t test	
P value	0,2869
P value summary	ns
Significantly different (P < 0.0	No
One- or two-tailed P value?	Two-tailed
t, df	t=1,119 df=11
How big is the difference?	
Mean ± SEM of column A	583,3 ± 519,7, n=8
Mean ± SEM of column B	4417 ± 4371, n=5
Difference between means	3834 ± 3426
95% confidence interval	-3706 to 11374
R squared (eta squared)	0,1022

data at Day 140	Osi	Osi-Afa
cpm/cm²	110	111
cpm/cm²	22,4	47,5
cpm/cm²	21900	140
cpm/cm²	32,7	27,3
cpm/cm²	21,9	55,8
cpm/cm²		22,4
cpm/cm²		42,5
cpm/cm²		4220

F test to compare variances	
F, DFn, Dfd	44,2, 4, 7
P value	<0,0001
P value summary	****
Significantly different (P < 0.0	Yes
