# Supplementary material for: Afatinib or Bevacizumab in combination with Osimertinib efficiently control tumor development in orthotopic murine models of non-small lung cancer
Source: PLoS One. 2024 Jun 27;19(6):e0304914. doi: 10.1371/journal.pone.0304914 (PMC11210880; doi:10.1371/journal.pone.0304914)
Supplement: S1 Table — Recapitulative tables of results of EC50 assays including number of experiments (n), means, standard deviation and standard error of mean. (PDF) [file pone.0304914.s016.pdf]

	Min cell number		A549		H1975		PC9		HCC827	
			wt	luc	wt	luc	wt	luc	wt	luc
topoisomerase II inhibitor	Etoposide	Number of values	3	3	4	3	3	3	4	4
		Mean	20,7	17,63	25,5	20,04	21,36	21,3	14,11	15,74
		Std. Deviation	5,603	6,748	7,793	4,396	3,072	3,702	8,658	11,09
		Std. Error of Mean	3,235	3,896	3,897	2,538	1,774	2,137	4,329	5,545
EGFR inhibitor	Erlotinib	Number of values	4	4	4	3	3	3	3	3
		Mean	52,75	57,85	42,82	39,4	64,21	70,23	13,5	11,46
		Std. Deviation	17,27	8,215	7,757	4,246	11,94	6,181	6,966	6,014
		Std. Error of Mean	8,635	4,107	3,878	2,451	6,895	3,569	4,022	3,472
EGFR inhibitor	Osimertinib	Number of values	3	3	3	3	3	3	3	3
		Mean	17,63	11,8	54,8	53,23	56,53	63,8	33,07	21,33
		Std. Deviation	5,229	2,696	9,991	14,4	8,57	9,296	5,445	4,362
		Std. Error of Mean	3,019	1,557	5,768	8,314	4,948	5,367	3,143	2,518
EGFR inhibitor	Afatinib	Number of values	3	3	3	3	3	3	3	3
		Mean	3,047	2,51	26,75	33,75	45,67	45	11,42	13,56
		Std. Deviation	0,5164	0,7499	8,972	0,7514	1,155	1	2,46	3,346
		Std. Error of Mean	0,2981	0,4329	5,18	0,4338	0,6667	0,5774	1,42	1,932
topoisomerase II inhibitor	Doxorubicin	Number of values	3	3	3	2	3	3	2	3
		Mean	16,26	9,51	23,21	21,68	20,34	19,56	17,13	22,67
		Std. Deviation	6,032	4,929	6,881	0,8518	2,977	4,829	6,972	7,982
		Std. Error of Mean	3,483	2,846	3,973	0,6023	1,719	2,788	4,93	4,608
platin salt	Cisplatine	Number of values	4	4	4	3	3	3	4	4
		Mean	16,71	11,64	29,4	28,07	25,34	26,14	17,15	17,87
		Std. Deviation	3,412	4,575	2,709	3,273	2,303	3,335	5,495	4,407
		Std. Error of Mean	1,706	2,287	1,355	1,89	1,33	1,926	2,748	2,203
platin salt	Carboplatin	Number of values	3	3	3	3	3	3	3	3
		Mean	27,03	29,3	46,33	39,63	23,27	22,9	33,7	41,23
		Std. Deviation	4,051	3,292	3,177	2,702	1,861	4,993	5,587	10,16
		Std. Error of Mean	2,339	1,901	1,834	1,56	1,074	2,883	3,225	5,867
DNA/RNA synthesis inhibitor	5FU	Number of values	4	4	4	4	4	4	4	4
		Mean	32,33	30,38	64,45	63,65	54,9	52,75	60,58	57,9
		Std. Deviation	3,919	3,825	1,898	3,291	4,641	3,77	7,417	4,217
		Std. Error of Mean	1,959	1,912	0,9491	1,645	2,321	1,885	3,708	2,108
Microtubule polymer stabilizer	Paclitaxel	Number of values	3	3	4	3	4	4	4	4
		Mean	28,53	21,03	26,81	22,88	13,6	11,71	16,64	16,77
		Std. Deviation	6,32	5,134	4,118	3,168	7,739	5,94	6,78	7,017
		Std. Error of Mean	3,649	2,964	2,059	1,829	3,869	2,97	3,39	3,509
c-Met and ALK inhibitor	Crizotinib	Number of values	3	3	3	3	3	3	3	3
		Mean	8,065	8,677	13,81	14,21	1,114	1,728	8,248	6,235
		Std. Deviation	9,731	9,577	10,58	12,69	0,3797	0,711	7,471	4,906
		Std. Error of Mean	5,618	5,529	6,111	7,329	0,2192	0,4105	4,314	2,833
ATM kinase inhibitor	AZD0156	Number of values	3	3	3	3	3	3	3	3
		Mean	12,56	11,59	19,94	19,09	7,162	8,66	19,1	18,08
		Std. Deviation	13,21	12,42	13,97	9,314	6,182	5,498	9,4	8,023
		Std. Error of Mean	7,625	7,171	8,064	5,377	3,569	3,174	5,427	4,632
ATM kinase inhibitor	KU55933	Number of values	3	3	3	3	3	3	3	3
		Mean	6,1	3,067	5,467	4,433	2,667	2,6	0,9667	1,2
		Std. Deviation	1,997	0,9452	2,71	0,9713	1,419	0,8	0,5859	0,8
		Std. Error of Mean	1,153	0,5457	1,565	0,5608	0,8192	0,4619	0,3383	0,4619
ATR kinase inhibitor	AZD6738	Number of values	3	3	3	3	3	3	3	3
		Mean	27,1	27,7	38,9	30,83	29	25,27	30,67	32,67
		Std. Deviation	5,285	6,643	18,93	18,75	2,623	6,979	8,752	6,261
		Std. Error of Mean	3,051	3,835	10,93	10,82	1,514	4,029	5,053	3,615
HDAC inhibitor	Panobinostat	Number of values	3	3	3	3	3	3	3	3
		Mean	26,01	24,8	29,78	27,91	30,15	31,1	35,75	34,09
		Std. Deviation	6,157	1,424	1,009	3,948	2,652	3,256	3,523	6,205
		Std. Error of Mean	3,554	0,8221	0,5825	2,279	1,531	1,88	2,034	3,583
HDAC inhibitor	Vorinostat	Number of values	3	3	3	3	3	3	3	3
		Mean	8,818	11,03	13,33	14,91	10,19	11,41	13,02	13,8
		Std. Deviation	0,7294	1,469	1,914	0,8788	0,953	1,201	1,27	3,499
		Std. Error of Mean	0,4211	0,8479	1,105	0,5074	0,5502	0,6934	0,7333	2,02
HDAC inhibitor	Belinostat	Number of values	3	3	3	3	3	3	3	3
		Mean	26,18	25,52	25,11	23,98	23,89	23,67	29,78	30,54
		Std. Deviation	5,037	0,2382	1,205	5,511	1,659	1,902	2,158	0,06899
		Std. Error of Mean	2,908	0,1375	0,6956	3,182	0,9577	1,098	1,246	0,03983
HDAC inhibitor	Droxinostat	Number of values	3	3	3	3	3	3	3	3
		Mean	35,18	32,4	38,55	31,7	25,37	28,39	30,37	30,89
		Std. Deviation	14,51	6,233	6,706	7,697	2,65	1,837	1,109	2,156
		Std. Error of Mean	8,379	3,598	3,871	4,444	1,53	1,061	0,6402	1,245
HDAC3 inhibitor	RGFP966	Number of values	3	3	3	3	3	3	3	3
		Mean	27,32	30,89	30,2	28,33	30,32	29,28	30,04	27,06
		Std. Deviation	1,379	7,342	2,071	1,496	0,03464	1,44	2,207	2
		Std. Error of Mean	0,7961	4,239	1,195	0,8638	0,02	0,8312	1,274	1,155
IRE1a endoribonuclease domain inhibitor	Mkc3946	Number of values	3	3	3	3	3	3	3	3
		Mean	21,6	23,27	30,97	23,73	10,2	8,933	10,43	7,133
		Std. Deviation	12,31	10,84	8,92	8,6	0,9644	1,692	7,049	5,262
		Std. Error of Mean	7,106	6,256	5,15	4,965	0,5568	0,977	4,07	3,038
PARP1/2 inhibitor	Olaparib	Number of values	3	3	3	3	3	3	3	3
		Mean	29,7	28,9	57,43	55,3	45,03	45,27	44,73	43,87
		Std. Deviation	9,718	10,19	1,742	3,65	2,454	4,562	6,274	2,757
		Std. Error of Mean	5,611	5,886	1,006	2,107	1,417	2,634	3,622	1,592
Wee1 inhibitor	MK1775	Number of values	3	3	3	3	3	3	3	3
		Mean	7,677	7,513	11,77	9,267	3,422	4,189	9,878	11,3
		Std. Deviation	2,468	3,023	2,808	0,4737	0,2035	0,8699	1,901	2,18
		Std. Error of Mean	1,425	1,745	1,621	0,2735	0,1175	0,5022	1,097	1,259
