# Supplementary material for: Afatinib or Bevacizumab in combination with Osimertinib efficiently control tumor development in orthotopic murine models of non-small lung cancer
Source: PLoS One. 2024 Jun 27;19(6):e0304914. doi: 10.1371/journal.pone.0304914 (PMC11210880; doi:10.1371/journal.pone.0304914)
Supplement: S2 Table — Recapitulative tables of results of Min for EC50 assays including number of experiments (n), means, standard deviation and standard error of mean. (PDF) [file pone.0304914.s017.pdf]

	EC50		A549		H1975		PC9		HCC827	
			wt	luc	wt	luc	wt	luc	wt	luc
topoisomerase II inhibitor	Etoposide	Number of values		3	4	4	4	4	4	4
		Mean	0,413	0,273	0,258	0,178	0,900	1,275	0,183	0,178
		Std. Deviation	0,344	0,142	0,184	0,169	0,141	0,310	0,146	0,097
		Std. Error of Mean	0,199	0,082	0,092	0,085	0,071	0,155	0,073	0,048
EGFR inhibitor	Erlotinib	Number of values	4	4	4	4	3	3	3	3
		Mean	11,1	10,78	7,875	7	0,00595	0,008933	0,003667	0,0005003
		Std. Deviation	2,043	1,461	2,78	2,582	0,005346	0,009432	0,005507	0,0008657
		Std. Error of Mean	1,021	0,7307	1,39	1,291	0,003087	0,005446	0,00318	0,0004998
EGFR inhibitor	Osimertinib	Number of values	3	3	3	3	3	3	3	3
		Mean	2,933	2,367	0,0029	0,0032	0,002333	0,002	0,001367	0,001567
		Std. Deviation	0,5508	0,8963	0,0001732	0,0008	0,0005774	0	0,0005686	0,0004041
		Std. Error of Mean	0,318	0,5175	0,0001	0,0004619	0,0003333	0	0,0003283	0,0002333
EGFR inhibitor	Afatinib	Number of values	3	3	3	3	3	3	3	3
		Mean	3,047	2,51	0,1277	0,1686	0,0001967	0,000098	0,0006	0,0005933
		Std. Deviation	0,5164	0,7499	0,01626	0,04067	0,000125	2,828E-06	0,00014	0,0001115
		Std. Error of Mean	0,2981	0,4329	0,009387	0,02348	0,00007219	0,000002	0,00008083	0,00006438
topoisomerase II inhibitor	Doxorubicin	Number of values	3	3	3	3	3	3	3	3
		Mean	0,0190	0,0233	0,0137	0,0097	0,0400	0,0500	0,0100	0,0097
		Std. Deviation	0,0102	0,0058	0,0055	0,0100	0,0000	0,0000	0,0000	0,0006
		Std. Error of Mean	0,0059	0,0033	0,0032	0,0058	0,0000	0,0000	0,0000	0,0003
platin salt	Cisplatine	Number of values	4	4	4	4	4	4	4	4
		Mean	3,075	3,85	3,05	3,3	0,6675	0,7845	3,3	0,6675
		Std. Deviation	0,8302	1,012	0,8226	1,961	0,1044	0,2351	0,8124	0,1044
		Std. Error of Mean	0,4151	0,5058	0,4113	0,9806	0,05218	0,1175	0,4062	0,05218
platin salt	Carboplatin	Number of values	3	3	3	3	3	3	3	3
		Mean	18,33	17,33	13,33	16,67	9,667	13	14,67	20
		Std. Deviation	2,517	3,786	1,155	1,528	2,517	2,646	4,726	6,245
		Std. Error of Mean	1,453	2,186	0,6667	0,8819	1,453	1,528	2,728	3,606
DNA/RNA synthesis inhibitor	5FU	Number of values	4	4	3	4	4	4	4	3
		Mean	0,5875	0,54	0,9133	1,1	1,57	1,778	0,84	1,087
		Std. Deviation	0,07455	0,07789	0,9416	0,8832	0,3535	0,8246	0,2758	0,6313
		Std. Error of Mean	0,03728	0,03894	0,5436	0,4416	0,1767	0,4123	0,1379	0,3645
Microtubule polymer stabilizer	Paclitaxel	Number of values	3	3	3	3	3	3	3	3
		Mean	0,00337	0,00267	0,00212	0,00160	0,00025	0,00030	0,00300	0,00267
		Std. Deviation	0,00055	0,00058	0,00153	0,00122	0,00009	0,00010	0,00100	0,00153
		Std. Error of Mean	0,00032	0,00033	0,00088	0,00070	0,00005	0,00006	0,00058	0,00088
c-Met and ALK inhibitor	Crizotinib	Number of values	3	3	3	3	3	3	3	3
		Mean	1,8	1,767	2,867	2,8	3,2	3,867	3,033	3,267
		Std. Deviation	0,4583	0,6351	1,55	1,4	1,179	0,5132	0,1528	0,3512
		Std. Error of Mean	0,2646	0,3667	0,895	0,8083	0,6807	0,2963	0,08819	0,2028
ATM kinase inhibitor	AZD0156	Number of values	3	3	3	3	3	3	3	3
		Mean	4,567	3,7	4,3	4,4	6,967	6,567	3,967	3,4
		Std. Deviation	1,872	1,735	1,3	1,311	2,205	2,542	1,026	0,8
		Std. Error of Mean	1,081	1,002	0,7506	0,7572	1,273	1,468	0,5925	0,4619
ATM kinase inhibitor	KU55933	Number of values	3	3	3	3	3	3	3	3
		Mean	2,967	3,133	2,9	2,233	3	3	1,933	2,1
		Std. Deviation	0,05774	0,2309	0,1732	0,3215	0	0	0,4041	0,5568
		Std. Error of Mean	0,03333	0,1333	0,1	0,1856	0	0	0,2333	0,3215
ATR kinase inhibitor	AZD6738	Number of values	3	3	3	3	3	3	3	3
		Mean	1,133	1,37	7,7	13,53	3,077	2,767	1,707	1,567
		Std. Deviation	0,3215	0,2524	5,231	7,068	0,06807	0,1528	0,1102	0,1528
		Std. Error of Mean	0,1856	0,1457	3,02	4,081	0,0393	0,08819	0,0636	0,08819
HDAC inhibitor	Panobinostat	Number of values	3	3	3	3	3	3	3	3
		Mean	0,01133	0,01233	0,012	0,013	0,01033	0,01	0,008667	0,006333
		Std. Deviation	0,001528	0,001155	0,001	0	0,004041	0,001	0,002082	0,003215
		Std. Error of Mean	0,0008819	0,0006667	0,0005774	0	0,002333	0,0005774	0,001202	0,001856
HDAC inhibitor	Vorinostat	Number of values	3	3	3	3	3	3	3	3
		Mean	2,1	1,767	2,167	2,033	1,367	1,467	2,067	1,7
		Std. Deviation	0,4	0,2082	0,3215	0,1528	0,1155	0,05774	0,5859	0,3
		Std. Error of Mean	0,2309	0,1202	0,1856	0,08819	0,06667	0,03333	0,3383	0,1732
HDAC inhibitor	Belinostat	Number of values	3	3	3	3	3	3	3	3
		Mean	0,05233	0,05333	0,03967	0,03533	0,02567	0,026	0,02767	0,01733
		Std. Deviation	0,006807	0,005774	0,009074	0,005033	0,004041	0,003606	0,007506	0,006429
		Std. Error of Mean	0,00393	0,003333	0,005239	0,002906	0,002333	0,002082	0,004333	0,003712
HDAC inhibitor	Droxinostat	Number of values	3	3	3	3	3	3	3	3
		Mean	96,67	87,33	80,67	99	68,67	73	43	27,67
		Std. Deviation	34,21	10,97	8,963	19,7	8,083	5,292	4,359	5,508
		Std. Error of Mean	19,75	6,333	5,175	11,37	4,667	3,055	2,517	3,18
HDAC3 inhibitor	RGFP966	Number of values	3	3	3	3	3	3	3	3
		Mean	20,6	19,07	25,6	28,77	23	20,17	10,17	6,933
		Std. Deviation	3,551	2,501	3,995	5,372	2,646	1,457	2,196	6,516
		Std. Error of Mean	2,05	1,444	2,307	3,102	1,528	0,8413	1,268	3,762
IRE1a endoribonuclease domain inhibitor	Mkc3946	Number of values	3	3	3	3	3	3	3	3
		Mean	103,3	87,33	49,67	37,33	51	54	38,67	42
		Std. Deviation	23,5	14,64	9,609	0,5774	7	4,583	8,622	4,583
		Std. Error of Mean	13,57	8,452	5,548	0,3333	4,041	2,646	4,978	2,646
PARP1/2 inhibitor	Olaparib	Number of values	3	3	3	3	3	3	3	3
		Mean	10	9,667	6	5,767	4,533	4,4	3,3	2,533
		Std. Deviation	6,083	3,512	1	1,328	1,286	1,039	0,6083	0,8083
		Std. Error of Mean	3,512	2,028	0,5774	0,7667	0,7424	0,6	0,3512	0,4667
Wee1 inhibitor	MK1775	Number of values	3	3	3	3	3	3	3	3
		Mean	1,333	1,133	1,367	1,467	0,9667	0,9667	0,9333	0,7333
		Std. Deviation	0,2082	0,1528	0,1155	0,1155	0,1528	0,05774	0,1155	0,05774
		Std. Error of Mean	0,1202	0,08819	0,06667	0,06667	0,08819	0,03333	0,06667	0,03333
